# Altered Adhesion and Migration of Human Mesenchymal Stromal Cells under Febrile Temperature Stress Involves NF-κβ Pathway

**DOI:** 10.1038/s41598-020-61361-z

**Published:** 2020-03-11

**Authors:** Ankita Sen, Malancha Ta

**Affiliations:** Indian Institute of Science Education and Research, Kolkata, India

**Keywords:** Extracellular matrix, Mesenchymal stem cells

## Abstract

Mesenchymal stromal cells (MSCs) are clinically beneficial for regenerative treatment of chronic inflammation and autoimmune disorders. However, to attain maximum efficacy from the transplanted MSCs, evaluation of its interaction with the microenvironment, becomes critical. Fever being an important hallmark of inflammation, we investigated the effect of febrile temperature stress on adhesion and migration of umbilical cord-derived MSCs. 40 °C-exposure altered cellular morphology with significant cell flattening, delayed cell-matrix de-adhesion response and slower migration of MSCs, accompanied by suppressed directionality ratio and cell trajectory. Corresponding to the observed changes, mRNA expression of extracellular matrix genes like *COLs* and *VTN* were upregulated, while matrix metalloproteinase *MMP-1*, showed a significant downregulation. NF-κβ pathway inhibition at 40 °C, led to reversal of gene expression pattern, cell spreading, de-adhesion dynamics and migration rate. Independent knockdown of p65 and p53 at 40 °C indicated inhibitory role of p65/p53/p21 axis in regulation of MMP-1 expression. P21 inhibits JNK activity, and JNK pathway inhibition at 40 °C resulted in further downregulation of *MMP-1*. Hence, our study provides the first evidence of cell migration getting adversely affected in MSCs under elevated temperature stress due to an inverse relationship between p65/p53/p21 and MMP1 with a possible involvement of the JNK pathway.

## Introduction

Found in the perivascular niche, mesenchymal stromal cells (MSCs) are regarded as promising source for cell-based treatment due to their medicinal effects at sites of disease, tissue injury and inflammation^1^. Though first isolated from bone marrow, MSCs have been successfully derived subsequently from multiple adult and fetal tissue sources, including the human umbilical cord which is a ‘younger’ and convenient alternative source of MSCs involving abundant availability and minimally invasive collection^2,3^. As multipotent progenitor cells, MSCs are capable of self renewal, multilineage differentiation, migration and homing and immunomodulation. In recent years, MSCs have been implicated specifically in immunetherapy, based on their ability to exert anti-inflammatory and immunomodulatory function, along with tissue regeneration, via paracrine and trophic mechanisms. However, existing MSC transplantation strategies are jeopardized by, massive cell death and poor engraftment *in vivo* following transplantation^4^. The resulting low survival rate hampers the clinical outcome of MSC based therapeutic applications. Moreover, MSCs harvested from different tissue sources could respond differently^5^, with respect to their immune properties, trophic factor secretion, migration and homing etc, to distinct microenvironmental stress for their clinical performance. The age of MSCs could also impact therapeutic efficacy significantly.

Inflammation is multifactorial and complex to understand, and the inflammatory milieu could be challenging due to the prevalence of oxidative stress, pro-inflammatory molecules, elevated temperature, etc. These microenvironment factors at the site of inflammation could alter some of the basic characteristics of MSCs and lead to cell loss. It is hence critical to scrutinize and identify the response of MSCs to the individual stress elements of an inflammatory milieu in order to maximize clinical benefits from MSC based therapy.

Febrile temperature response is a hallmark feature of inflammatory disease and infection^6^. However, the impact of febrile temperature stress on MSC properties has not been very extensively studied. In a previous study from our group, we had demonstrated G0/G1 cell cycle arrest along with a crosstalk between NF-κβ and p53 in MSCs subjected to physiological fever-like temperature^7^. Little is known about the effects of heat stress on the migration of MSCs. The success of MSC clinical applications relies on efficient migration and homing of MSCs to the target site following administration^8^. Hence, an important MSC property is their migration ability. As elevated temperature stress can be considered as a critical extracellular stimulus, here we assessed its impact on adhesion and migration of human Wharton’s jelly-MSCs (WJ-MSCs). WJ is the connective tissue surrounding the umbilical cord vessels, and a rich source of MSCs^2^.

On exposure to elevated temperature condition, WJ-MSCs attained a flattened morphology with significant increase in cell area and reduced migration rate with compromised directionality ratio and mean square displacement (MSD) of cell body. They exhibited stronger adherence to plastic surface with a significantly delayed de-adhesion response to trypLE. Correspondingly, an increase in the mRNA expression level of several ECM genes associated with adhesion, such as collagens (*COLs*) and Vitronectin (*VTN)*, and a downregulation of matrix metalloproteinases like *MMP1* and *ADAMTS1* were observed at 40 °C. Activation of NF-κβ pathway appeared to be responsible for the observed changes in gene expression, as siRNA mediated downregulation of NF-κβ resulted in a strong reversal effect on the gene expression pattern of the above markers. Moreover, a reversal effect on cell spread area, cell-matrix de-adhesion time constants and migration rate too was noted. Tumor suppressor protein p53 was upregulated at 40 °C, which in turn appeared to control the downregulation of *MMP1*, as confirmed by knock down studies using *P53* siRNA. This was possibly executed via p21. NF-κβ pathway inhibition also reversed the p53 and p21 inductions at 40 °C with a corresponding increase in *MMP1*. Additionally, as integrin mediated signalling is known to control gene expression, when we knocked down VTN, we noted a downregulation of p53 and p21 with a corresponding increased expression of *MMP1*. JNK signalling pathway inhibition at 40 °C led to further downregulation of *MMP1*, with p53/p21 maintained at high levels. To sum up, an inverse relationship between p53/p21 and MMP1 was established in WJ-MSCs at 40 °C, with a possible involvement of the JNK pathway.

## Results

### Impact of febrile temperature stress on cell size and migration of WJ-MSCs

**Figure 1:**
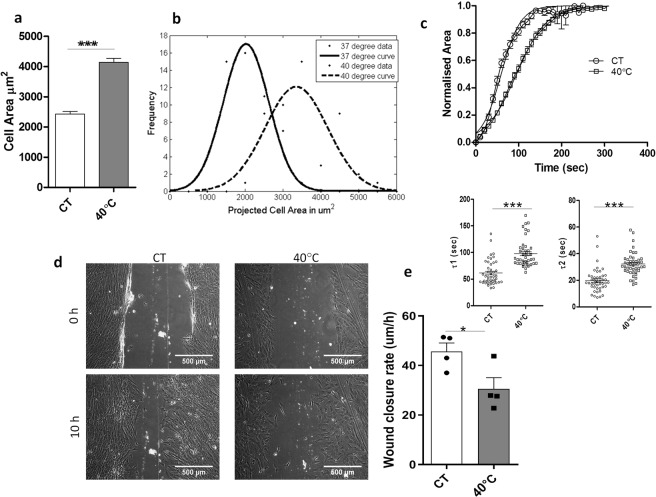
Cell spread area and migration of WJ-MSCs under elevated temperature stress. Quantification of cell area. Cell area was plotted from a total of 150 cells from 3 different biological samples (**a**) A representative Gaussian distribution plot from 50 cells to demonstrate comparison in cell area between control WJ-MSCs and WJ-MSCs cultured at 40 °C is shown (**b**). Effect of elevated temperature on time constants of retraction. WJ-MSCs were treated with TrypLE to sever cell-ECM contacts. With the help of time lapse imaging, time taken by the MSCs to contract to a rounded morphology was recorded. The normalized area-vs.-time data were fitted to a Boltzmann sigmoid equation to determine the time constants τ1 and τ2. Delayed de-adhesion yielding greater values for both the time constants are displayed (**c**). Comparison of scratch-induced migration between control and febrile temperature-treated WJ-MSCs. Confluent monolayer cultures of control and 40 °C-treated WJ-MSC cultures were wounded with a sterile pipette tip at 0 h. Images of migration post scratch assay were obtained at 10 h. Scale bar: 500 µm. Representative images from four independent biological samples (n = 4) are shown (**d**). Wound closure rate was quantified and compared between control WJ-MSCs and WJ-MSCs under febrile temperature stress (n = 4) (**e**). Each bar represents mean ± SEM (n ≥ 3). Student’s t-test, two-tailed, *represents p < 0.05, ***represents *p* < 0.0001.

As reported earlier^7^, there was a distinct morphological difference in WJ-MSCs under control vs febrile temperature conditions. At 40 °C, WJ-MSCs lost the characteristic spindle shape morphology, and adopted a more flattened appearance. Cell spread area was measured and compared between the two populations, and a cell size histogram was plotted in Fig. 1a,b (*p* < 0.0001).

The ISCT had proposed the physical adherence of MSCs to plastic surface as one of the minimal criteria for defining MSCs^9^. There was a stronger cell adherence to the tissue culture plastic surface by the 40 °C treated WJ-MSCs in comparison to control WJ-MSCs as indicated by cell dissociation time taken during TrypLE treatment. We next attempted to quantify and compare the de-adhesion dynamics following TrypLE action. The cell–matrix adhesive contacts were severed with TrypLE and the cellular retraction that preceded cell detachment was followed by live cell imaging. The 40 °C exposed WJ-MSCs took longer to retract and round up as compared to the control WJ-MSCs, as evident from right shift of the curve (Fig. 1c). The τ1 and τ2 values for control and 40 °C treated WJ-MSCs were 61.5 ± 3.4 s, 98.1 ± 3.8 s and 19.7 ± 1.3 s, 32.1 ± 1.3 s, respectively (*p* < *0.0001*) (Fig. 1c). Hence, elevated temperature stress altered de-adhesion dynamics of WJ-MSCs.

To investigate if the change in cell spread area at 40 °C was accompanied with a difference in migration rate or pattern, we first performed an *in vitro* scratch-induced wound healing assay. Cells were scraped from an area of confluent monolayer culture of WJ-MSCs grown under 40 °C and control condition respectively (Fig. 1d). Migration rate was assessed during wound closure. WJ-MSCs at 40 °C were slower to migrate and took longer to heal the wound as expressed by wound closure rate in Fig. 1e (*p* < 0.05).

### Impact of febrile temperature stress on migration of WJ-MSCs in real time

**Figure 2:**
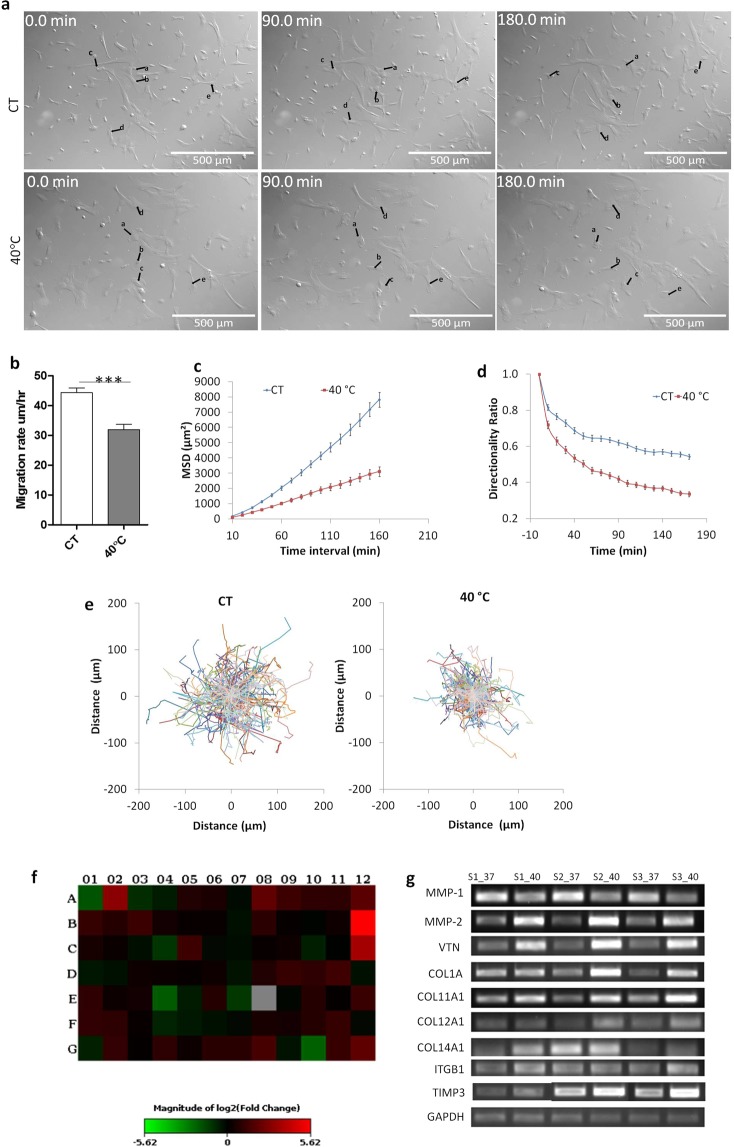
Migration and gene expression analysis of control and febrile temperature treated WJ-MSCs. Time lapse videos were captured of WJ-MSCs under control and febrile temperature conditions. Representative DIC still images were taken from the time-lapse videos of control WJ-MSCs and WJ-MSCs exposed to febrile temperature condition. Black arrows show the migration of a few randomly selected cells. Numbers on the images indicate time in minutes. Results are representative of three independent biological samples (n = 3). Scale bar: 500 µm (**a**). Comparison of migration speed between control WJ-MSCs and those under elevated temperature condition. Sample size used was 30 cells per sample and three different biological samples were used. Bars represent mean ± SEM. (Student’s t-test, two-tailed, ***represents *p* < 0.0001) (**b**). To further assess the irregular migration of febrile temperature treated WJ-MSCs, MSD of cell body (**c**), directionality ratio (**d**) and trajectories of cells (**e**) were calculated for n = 150 cells from three different biological samples. Changes in ECM and adhesion molecule gene expression in WJ-MSCs at 40 °C as demonstrated by PCR array. A Heat Map comparing ECM and adhesion molecule gene expression between control WJ-MSCs and WJ-MSCs cultured at 40 °C for 48 h is displayed. Genes with at least 2 fold differential expression have been considered (**f**). Semi-quantitative RT-PCR validation of a few randomly selected genes from the ECM and Adhesion Molecules RT^2^ profiler PCR Array was performed using cDNAs from three different sets of samples. *GAPDH* was used as an internal control (**g**). Full length gels are presented in Supplementary Figure S2.

To further confirm in real time the influence of elevated temperature on migration, WJ-MSCs were cultured at 40 °C for 48 h and a time lapse video was captured over a time frame of ~3 h (Fig. 2a,b and Supplementary Videos S1 and S2). While, control WJ-MSCs underwent typical robust migratory movement, the heat stressed WJ-MSCs exhibited slower migration which was further analysed by measuring migration rate, single cell trajectory, cell directionality and mean square displacement (MSD) over a period of 3 h using the DiPer software^10,11^. The heat stressed WJ-MSCs had significantly reduced migration rate (*p* < 0.0001) (Fig. 2b) along with a decrease in MSD (Fig. 2c) as compared to control WJ-MSCs. This was further supported by lesser spread area of cell trajectory of heat stressed WJ-MSCs as compared to control cells (Fig. 2e). The WJ-MSCs at 40 °C even displayed suppressed directionality ratio (Fig. 2d).

### Effect of elevated temperature stress on ECM and adhesion molecule gene expression

Since at elevated temperature of 40 °C, WJ-MSCs exhibited greater cell spread area and reduced migration rate, we attempted a large scale gene expression study using the Human Extracellular Matrix and Adhesion Molecules RT^2^ Profiler PCR Array. This array profiled the expression of 84 different genes associated with cell-cell and cell-matrix interactions (Supplementary Table 1). Genes with ≥2 fold change in expression between elevated temperature treated and the corresponding control WJ-MSCs were taken into consideration (Table 1). Out of the 84 different genes screened, many of the collagen family genes, such as *COL1A1, COL11A1, COL12A1, COL14A1, COL16A1* etc were found upregulated in WJ-MSCs cultured at 40 °C. Some of the other key genes upregulated were, *VTN*, which is an adhesive glycoprotein and a serum attachment and spreading factor^12^, *ADAMTS13* and some of the laminins. During the migration process, action of proteolytic enzymes such as matrix metalloproteinases (MMPs) is required to degrade the ECM^13^. Interestingly, *MMP1* was downregulated at 40 °C, while there was an upregulation of *MMP2* and *MMP3*, and also of *TIMP3*, which is an MMP inhibitor. A recent study showed MMP1 to be a direct inhibitory target of TIMP3^14^. A Heat Map of the data is demonstrated in Fig. 2f.

**Table 1:** Differentially expressed genes between control and febrile temperature-treated WJ-MSCs as established using the Human Extracellular Matrix & Adhesion Molecules RT² Profiler PCR Array.

*No*.	*Gene Symbol*	*Gene Description*	*Fold Difference*
*Genes upregulated in WJ-MSCs under elevated temperature condition compared to control condition*			
**1**	*ADAMTS 13*	ADAM metallopeptidase with thrombospondin type 1 motif, 13	8.4525
**2**	*COL11A1*	Collagen, type XI, alpha 1	4.2894
**3**	*COL12A1*	Collagen, type XII, alpha 1	2.5053
**4**	*COL14A1*	Collagen, type XIV, alpha 1	2.0276
**5**	*COL16A1*	Collagen, type XVI, alpha 1	4.0012
**6**	*COL1A1*	Collagen, type I, alpha 1	2.228
**7**	*COL5A1*	Collagen, type V, alpha 1	2.4347
**8**	*CTNND2*	Catenin (cadherin-associated protein), delta 2 (neural plakophilin-related arm-repeat protein)	49.1785
**9**	*ITGA1*	Integrin, alpha 1	2.5349
**10**	*ITGA8*	Integrin, alpha 8	12.4816
**11**	*ANOS1*	Kallmann syndrome 1 sequence	2.3055
**12**	*LAMA1*	Laminin, alpha 1	2.0177
**13**	*LAMA2*	Laminin, alpha 2	2.5668
**14**	*LAMB1*	Laminin, beta 1	2.035
**15**	*MMP2*	Matrix metallopeptidase 2 (gelatinase A, 72 kDa gelatinase, 72 kDa type IV collagenase)	2.347
**16**	*MMP3*	Matrix metallopeptidase 3 (stromelysin 1, progelatinase)	2.0808
**17**	*SPARC*	Secreted protein, acidic, cysteine-rich (osteonectin)	2.1966
**18**	*TGFBI*	Transforming growth factor, beta-induced, 68 kDa	2.1416
**19**	*TIMP3*	TIMP metallopeptidase inhibitor 3	2.9272
**20**	*VCAN*	Versican	2.2657
**21**	*VTN*	Vitronectin	4.1704
*Genes down-regulated in WJ-MSCs under elevated temperature condition compared to control condition*			
**1**	*ADAMTS1*	ADAM metallopeptidase with thrombospondin type 1 motif, 1	−3.6311
**2**	*ICAM1*	Intercellular adhesion molecule 1	−2.2417
**3**	*MMP1*	Matrix metallopeptidase 1 (interstitial collagenase)	−4.0059
**4**	*MMP12*	Matrix metallopeptidase 12 (macrophage elastase)	−2.3344
**5**	*VCAM1*	Vascular cell adhesion molecule 1	−4.2899

### RT-PCR validation of Array data

A few genes were randomly selected from the Human Extracellular Matrix and Adhesion Molecules RT^2^ Profiler PCR Array and subjected to semi-quantitative RT-PCR in order to validate the array data. Samples used were the pooled cDNA sets used in the array as well as individual cDNA samples comprising the pool. The differential gene expression pattern observed by semi-quantitative RT-PCR for *COL1A1, COL11A1, COL12A1, COL14A1, ITGB1, MMP1, MMP2, TIMP3* and *VTN* between elevated temperature treated and the corresponding control WJ-MSC samples, were in good agreement with the array data (Fig. 2g). Based on the altered adhesion and migration observed in the WJ-MSCs at 40 °C, we selected *MMP1, MMP2* and *VTN* from the panel for further analysis.

### Effect of NF-κβ inhibition on the expression of *MMP1*, *MMP2* and *VTN*

NF-κβ is a transcription factor playing a key role in diverse cellular processes such as inflammation, tumor progression and immune response^15^. We next wanted to determine if the NF-κβ signalling pathway was associated with the observed changes in mRNA expression levels *of MMP1, MMP2* and *VTN* under elevated temperature stress condition. To address this, WJ-MSCs were treated at 40 °C for 48 h with a specific inhibitor of NF-κβ pathway, Pyrrolidinedithiocarbamate (PDTC)^16^, which has dependence on zinc for its action. Although no direct effect of febrile temperature on zinc has been reported, lower level of zinc has been shown to be a triggering factor for developing febrile convulsions^17^.

**Figure 3:**
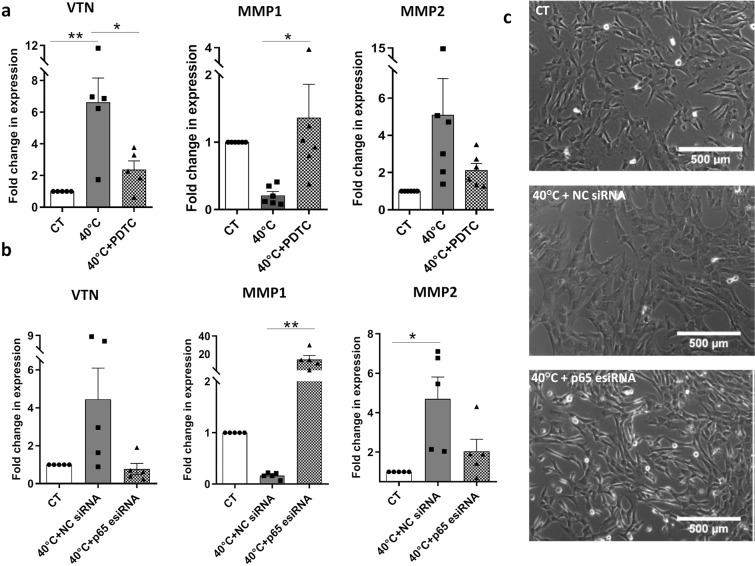
Reversal of gene expression and morphology following NF-κβ pathway inhibition. WJ-MSCs were treated with 700 nM of NF-κβ inhibitor PDTC at 40 °C for 48 h, and the expressions of *MMP1, MMP2* and *VTN* genes were detected by qRT-PCR. Values were normalized to GAPDH. Results are representative of six independent biological samples (n = 6). *Represents p < 0.05, **represents *p* < 0.01 (**a**). WJ-MSCs were transfected with negative control (NC) siRNA or p65-targeted siRNA at 40 °C for 48 h and mRNA expression levels of *MMP1, MMP2* and *VTN* were determined by qRT-PCR analysis. Values were normalized to *GAPDH*. Results are representative of five independent biological samples (n = 5). Bar represents mean ± SEM. *Represents p < 0.05, **represents *p* < 0.01 (**b**). Morphological changes were observed in WJ-MSCs cultured at 40 °C for 48 h as compared to control WJ-MSCs. p65 knockdown using esiRNA at 40 °C led to reversal in morphology towards spindle shape of control WJ-MSC culture. Representative phase contrast images from five independent WJ-MSC cultures are displayed (n = 5). Scale bar: 500 µm (**c**).

The changes in the gene expression of *MMP1, MMP2* and *VTN*, noted at 40 °C, were reversed by the treatment with PDTC (Fig. 3a), as there was an increase in *MMP1* expression (*p* < 0.05) and decrease in *VTN* (*p* < 0.05) and *MMP2* (not significant) gene expression.

**Figure 4:**
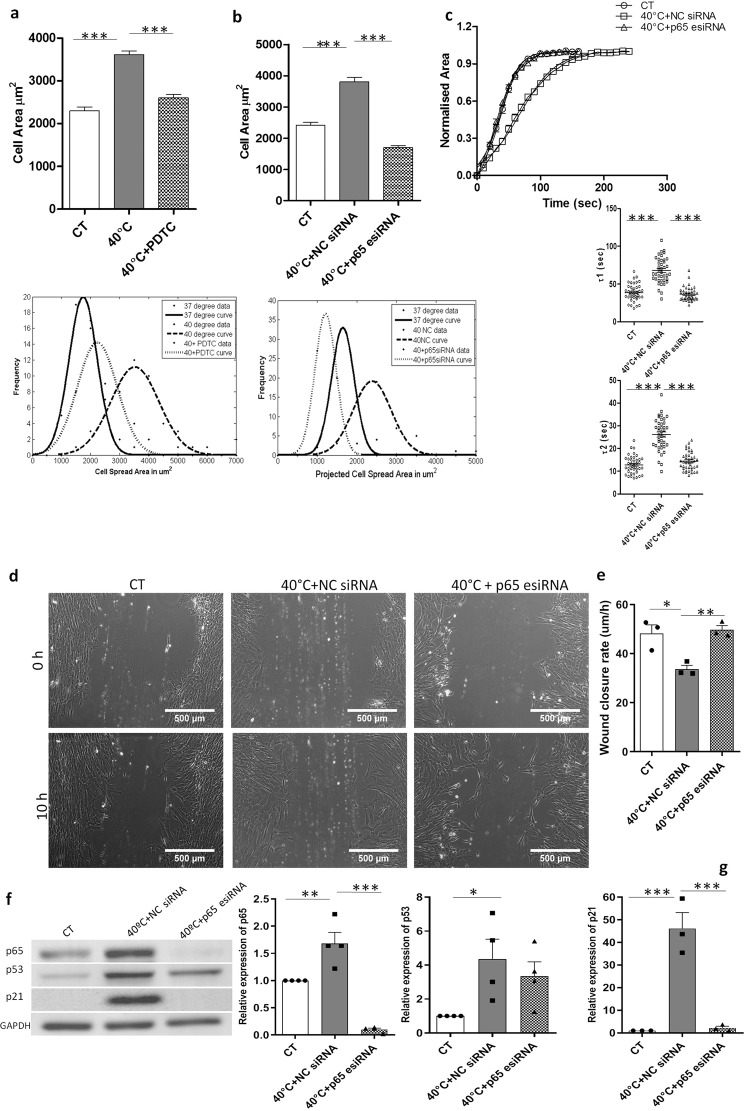
Reversal of cell spread area, de-adhesion dynamics and migration following NF-κβ pathway inhibition. WJ-MSCs were treated with 700 nM PDTC at 40 °C, cell area was determined, and plotted against corresponding control cultures. A sample size of 150 total cells from three different biological samples was used. A representative Gaussian distribution plot from 50 cells to depict cell area comparison is shown, ***represents *p* < 0.0001 (**a**). Similarly, cell area was determined for WJ-MSCs transfected with p65-targeted esiRNA or NC siRNA at 40 °C for 48 h and plotted against control WJ-MSC samples. A sample size of 200 total cells, from four different biological samples, was used, ***represents *p* < 0.0001. A representative Gaussian distribution plot from 50 cells to depict cell area comparison is shown. (**b**). De-adhesion dynamics of p65 esiRNA transfected WJ-MSCs. Differences in τ1 and τ2 between negative control siRNA and p65 esiRNA treated cells were significant, ***represents *p* < 0.0001 (**c**). Comparison of scratch wound- induced migration between control WJ-MSCs and WJ-MSCs transfected with NC siRNA or p65-targeted esiRNA at 40 °C was undertaken. Representative images are shown (n = 3) (**d**). Wound closure rate in µm/h was estimated and compared between the three conditions (n = 3), *represents p < 0.05, **represents *p* < 0.01 (**e**). There was a reduction in p65 protein level on transfecting WJ-MSCs with p65-targeted esiRNA as detected by Western blot. Reduced expression of p53 and p21 proteins was noted too in the p65 esiRNA targeted WJ-MSC samples. GAPDH was used as a loading control (**f**). Band intensities were quantified relative to GAPDH and plotted (n = 4), *represents p < 0.05, **represents *p* < 0.01, *** represents *p* < 0.001 (**g**). Full length blots are presented in Supplementary Figure S3.

To achieve a more specific inhibition of NF-κβ pathway, we next knocked down the p65 subunit of NF-κβ with *p65* targeted endoribonuclease-prepared siRNA (esiRNA) at 40 °C. The transfected cells were first assayed for reduction in p65 protein by western blot analysis (Fig. 4f,g) (*p* < 0.001). The mRNA expression levels of *VTN, MMP1* and *MMP2* were investigated in p65 esiRNA transfected WJ-MSCs and again there was a strong reversal in the expression of *MMP1 (p* < *0.01)*, while *MMP2* and *VTN* showed a marked reduction though not significant as compared to WJ-MSCs at 40 °C transfected with negative control siRNA (NC siRNA) (Fig. 3b). There was also a reversal in the gene expression of *COL1A* and *COL12A1*, but no change in *COL11A1*, under both PDTC (*p* < 0.05) and p65 targeted esiRNA treatments (Supplementary Fig. S1).

We next wanted to assess the involvement of NF-κβ signalling pathway, if any, in the morphology change, altered de-adhesion dynamics and impaired migration of WJ-MSCs observed at 40 °C. Transfection of WJ-MSCs with p65 targeted esiRNA at 40 °C resulted in morphological alterations, with the cells resuming spindle shaped phenotype (Fig. 3c). There was reversal of cell spread area toward that of control WJ-MSCs, with both PDTC and p65 siRNA treatment (Fig. 4a,b (*p* < 0.0001). Interestingly, the cell de-adhesion delay observed at 40 °C was also reversed significantly with p65 esiRNA treatment, with time constants of τ1 = 36.5 ± 1.4 s and τ2 = 14.3 ± 0.6 s (Fig. 4c). The time constants τ1 and τ2 were 67.9 ± 2.6 s and 26.2 ± 1.1 s for negative control esiRNA transfected WJ-MSCs at 40 °C. Hence, NF- κβ pathway inhibition altered the de-adhesion dynamics of WJ-MSCs under elevated temperature stress. Furthermore, the impact of NF-κβ pathway inhibition was studied on cell migration activity of WJ-MSCs at 40 °C. The p65 esiRNA transfected WJ-MSCs showed a significantly increased migratory activity towards filling the gap made by a scratch as compared to negative control siRNA transfected WJ-MSCs (Fig. 4d,e).

### Role of p53 in heat stress induced downregulation of *MMP1*

**Figure 5:**
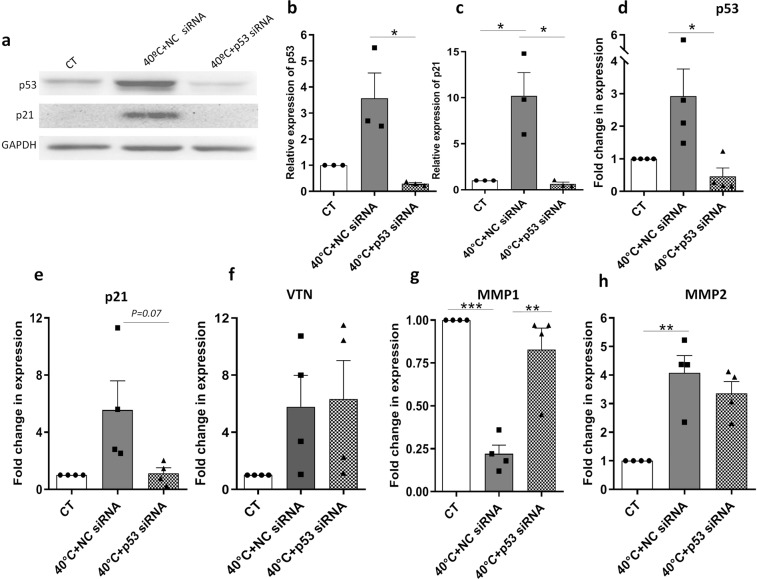
Impact of p53 knockdown on gene expression of *MMP1, MMP2* and *VTN*. WJ-MSCs were transfected with a NC siRNA or a *p53*-targeted siRNA for 48 h at 40 °C. The siRNA mediated transfection efficiency for p53 was determined by Western blotting. Protein expression of p21 too was detected by Western blot. GAPDH was used as a loading control (**a**). Band densities of p53 and p21 proteins were quantified relative to GAPDH and plotted (n = 3), *represents p < 0.05 (b, c). qRT-PCR analysis in WJ-MSC transfectant cells. Effect of p53 knockdown with specific siRNA, on the gene expressions of *p53* (**d**), *p21* (**e**), *VTN* (**f**), *MMP1* (**g**) and *MMP2* (**h**), was demonstrated by qRT- PCR. WJ-MSCs transfected with a NC siRNA served as a negative control, while GAPDH was used as an endogenous control. Results are representative of three independent biological samples (n = 3). Bar denotes mean ± SEM, *represents *p* < 0.05, **represents *p* < 0.01, ***represents *p* < 0.001. Full length blots are presented in Supplementary Figure S3.

Tumor suppressor gene, TP53, also called the guardian of the genome, coordinates a wide range of cellular processes in response to different cellular stress factors^18^. It is well established by now that other than its cell cycle related roles, p53 is also involved in cell migration and invasion regulation in cancer cell metastasis. P53 has been reported to oppose epithelial–mesenchymal transition (EMT) and cell migration^19^. In addition, in a few earlier reports, p53 has been demonstrated to downregulate *MMP1* gene expression. In our previous study, we had demonstrated an increase in p53 protein expression and its nuclear translocation in WJ-MSCs exposed to 40 °C^7^. Here too we noted an increase in both mRNA (Fig. 5d) (not significant) and protein expression levels of p53 at 40 °C (Fig. 4f,g) (significant), which was reversed on esiRNA mediated knockdown of p65 at 40 °C (Fig. 4f,g) (not significant). As p53 protein acts as a transcription factor, to investigate its involvement in gene expression of *MMP1, MMP2* and *VTN*, p53 was inhibited using siRNA transfection and the effect of its abrogation was studied in WJ-MSCs at 40 °C. *p53* siRNA transfected cells expressed reduced levels of both p53 mRNA and protein (Fig. 5a,b,d) (*p* < 0.05). Also, when compared with negative control siRNA transfected cells, p53-targeted siRNA transfected WJ-MSCs showed a significant increase in the mRNA expression of *MMP1* (Fig. 5g), whereas *VTN* and *MMP2* mRNA expressions did not alter much as presented in Fig. 5f,h, respectively. P21, a downstream target of p53 which was induced at both mRNA (Fig. 5e) (not significant) and protein levels at 40 °C (Fig. 5a,c) (*p* < 0.05) showed a marked reduction on inhibiting p53 (Fig. 5a,c,e), the difference being significant at the protein level. A previous study had shown a vital role for p21in suppressing MMPs^20^. Consistently, in our results we noted a down regulation of p21 with a simultaneous upregulation of *MMP1*on inhibiting p53.

### VTN regulates the expression of p53, p21 and *MMP1*

**Figure 6:**
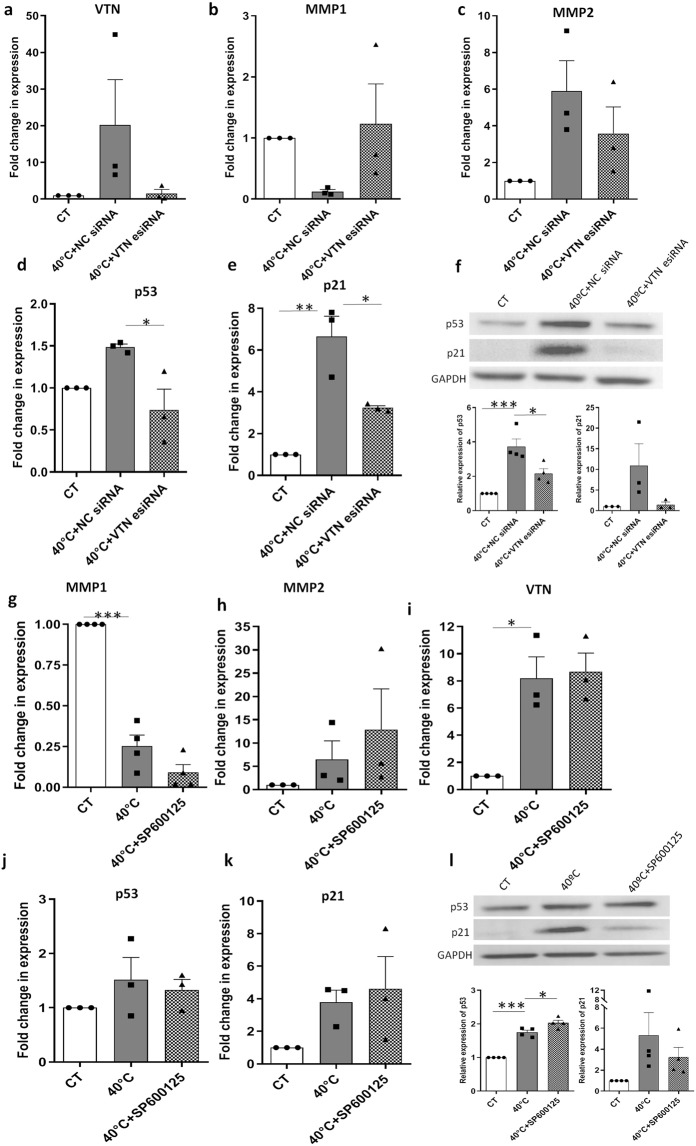
P53 regulation by VTN and the effect of JNK pathway inhibition in WJ-MSCs at 40 °C. WJ-MSCs were transfected with a NC siRNA or a VTN-targeted esiRNA for 48 h at 40 °C. qRT-PCR analysis of WJ-MSC transfectant cells. The siRNA mediated transfection efficiency for *VTN* was determined by qRT-PCR (**a**). Effect of VTN knockdown, on the gene expression of *MMP1* (**b**), *MMP2* (**c**), *p53* (**d**) and *p21* (**e**), was demonstrated by qRT-PCR. WJ-MSCs transfected with a NC siRNA served as a negative control, while *GAPDH* was used as an endogenous control. Results are representative of at least three independent biological samples (n ≥ 3). Bar represents mean ± SEM, *represents *p* < 0.05, **represents *p* < 0.01. Cell lysates were blotted to detect the protein levels of p53 and p21, with GAPDH used as loading control. Results are representative of at least 3 independent biological samples (n ≥ 3). Band densities were quantified relative to GAPDH and plotted, *represents *p* < 0.05, ***represents *p* < 0.001 (**f**). Effect of JNK pathway inhibition in WJ-MSCs at 40 °C. qRT-PCR detection and comparison between control and 40 °C exposed WJ-MSCs, in the presence and absence of 10 µM SP 600125, for *MMP1* (**g**), *MMP2* (**h**), *VTN* (**i**), *p53* (**j**) and *p21* (**k**) are shown. *GAPDH* was used as an endogenous control. Results are representative of at least three independent biological samples (n ≥ 3). Bar represents mean ± SEM, *represents *p* < 0.05, ***represents *p* < 0.0001. Cell lysates were blotted to detect the level of p53 and p21 proteins. GAPDH was used as a loading control. Band densities were quantified relative to GAPDH and plotted (n = 3), * represents *p* < 0.05, *** represents *p* < 0.0001 (**l**). Full length blots are presented in Supplementary Figure S3.

For most biological functions, under both normal and pathological conditions, integrin based signalling is critical. Besides their mechanical role in anchorage, integrins can transmit chemical signals into the cells which further control cellular responses such as survival, differentiation, migration etc^21^. Since integrin signalling can alter gene expression patterns, and as VTN is an ECM component which associates with the integrins, we wanted to explore if VTN regulates the expression of MMPs in the WJ-MSCs at 40 °C. On *VTN* esiRNA transfection, WJ-MSCs expressed reduced level of *VTN* mRNA (Fig. 6a) (not significant). *VTN* esiRNA transfected WJ-MSCs expressed higher level of *MMP1*, though difference was not significant (Fig. 6b). There was a reduction in *MMP2* expression, difference was not significant (Fig. 6c). Furthermore, there was a reduction in both the mRNA and protein levels of p53 (Fig. 6d,f) (*p* < 0.05) and its downstream effector, p21(Fig. 6e,f).

### Impact of JNK pathway inhibition on *MMP1* expression

JNK pathway’s involvement in the control of cell motility and adhesion is established. Studies in mammalian cell lines have demonstrated JNK activated transcription factor AP1 as regulator of MMP gene expression^22^. Since a previous study demonstrated that p21 suppressed the expression of IL6 and MMP1expression in synovial fibroblasts by inhibiting the JNK/AP1 pathway^19^, we tested the role of JNK pathway, if any, on expression of MMP1in WJ-MSCs at 40 °C. When we inhibited JNK pathway using 10 µM of small molecule inhibitor SP 600125^23^ at 40 °C, *MMP1* expression was further downregulated as compared to untreated WJ-MSCs at 40 °C (Fig. 6g), though the difference was not significant. No specific trend was noted for *MMP2* or *VTN* (Fig. 6h,i) expression, respectively. In the presence of SP600125, not much change was detected in the mRNA or protein levels of p53 and p21 as compared to untreated samples at 40 °C (Fig. 6j–l). Their expressions were maintained at a higher level as compared to control WJ-MSCs.

## Discussion

As febrile response is a hallmark of inflammatory diseases, here we evaluated the biological effect of heat stress, within physiological fever range, on adhesion and migration of WJ-MSCs.

Exposure to 40 °C for 48 h led to changes in cellular morphology in WJ-MSCs while simultaneously causing reduced migration, where parameters such as MSD, directionality ratio and single cell trajectory got negatively affected. Using de-adhesion assay, WJ-MSCs at 40 °C were also shown to have delayed detachment from tissue culture plastic surface as compared to control WJ-MSCs, suggesting impairment of cellular de-adhesion response at 40 °C.

There exists a fine tensional balance between cytoskeleton of the cell and the rigidity of the ECM, which could play regulatory roles in a variety of cellular processes such as cell migration, cell shape, cell fate choices etc.^24^. Our ECM and Adhesion molecule PCR array result revealed a higher expression of a large number of ECM and adhesion molecule genes, such as various collagen family genes, VTN and some integrins in WJ-MSCs at 40 °C. CTNND2, an adherens junction protein which has been reported to have a role in cell motility and being expressed early during neuronal development^25^, was found markedly upregulated in the array data. The α8 integrin, which is expressed on mesenchymal cells and dimerizes with β1 integrin chain to serve as a receptor for fibronectin, VTN, tenacin etc^26^, was also found to be upregulated. However, during the validation using semi-quantitative RT-PCR with individual samples sets, these two genes did not show a strong increase in expression in the 40 °C treated samples. As a result, they were not pursued further. Other than these two, a high level of reproducibility between the PCR array data and the semi-quantitative RT-PCR validation for a good number of randomly selected genes, confirmed the fidelity of our array data.

*VTN, MMP1* and *MMP2* were selected to further elucidate the increased adhesion and migration impairment observed in WJ-MSCs at 40 °C. Though there are contradictory reports regarding the role of MMP2 in MSC migration, MMP1 has been shown to be important for MSC migration^27^. Other than their migratory role, MMPs have also been reported to be upregulated during inflammation and can act as modulators of inflammation.

We noted a decrease in *MMP1* mRNA level in a time-dependent manner while *MMP2* and *VTN* showed a time-dependent increase in their mRNA expression levels, over a period of 72 h at 40 °C as detected by qRT-PCR (Supplemental Fig. S1).

Previously, a study in HUVECs identified that NF-κβ signalling pathway was essential for resistance to apoptosis in response to heat stress^28^. We had earlier demonstrated a cell cycle arrest of WJ-MSCs at 40 °C along with an induction of p53 which was reversed on inhibiting NF-κβ pathway^7^. In the present research, we explored the role of NF-κβ pathway, if any, in the change in gene expression of *MMP1, MMP2* and *VTN* at 40 °C. NF-κβ binding site had been identified in the MMP1 promoter region. Earlier reports had shown activated NF-κβ to play an important role in inducing MMP1 expression^29^. In contrast, we found NF-κβ pathway responsible for downregulation of *MMP1* in WJ-MSCs at 40 °C, as on inhibiting this pathway, there was upregulation of *MMP1* gene expression. And, there was a decrease in the expression of *MMP2* and *VTN*. In support, NF-κβ had been shown to regulate the expression of *MMP2*, where NF-κβ inhibitors downregulated *MMP2* expression^30^. A regulatory role for NF-κβ in ECM protein production, including fibronectin and VTN had been also shown previously^31^. In addition, NF-κβ pathway inhibition had a reversal effect on cell spreading, adhesion and migration. Interestingly, when control WJ-MSCs at 37 °C were treated with PDTC/p65 esiRNA, the change in gene expression pattern followed a similar trend, with an increase in *MMP1* and decrease in *MMP2* and *VTN* (Supplementary Fig. S1).

NF-κβ and p53 are transcription factors which play pivotal regulatory roles in determining cellular fate, and cross talk between them at multiple levels has been acknowledged during different stages of tumorogenesis, immune surveillance and metastasis^32^. These interactions, however, are mostly cellular context specific. On verifying the level of p53 protein in WJ-MSCs at 40 °C, we noted an increase in the expression of both p53 and its downstream target, p21, which on inhibiting NF-κβ pathway, showed a decrease. P53 is a transcription factor which regulates multiple target genes with diverse biological functions. Knocking down of p53, with a corresponding decrease in p21, resulted in increased expression of *MMP1*. *MMP1* expression had been shown to be inhibited by p53 in earlier reports^33^. Therefore, an inverse relation between p53/p21 and *MMP1* in WJ-MSCs at 40 °C was established. *VTN* and *MMP2* did not get affected by p53 knockdown.

A previous report demonstrated a role of activated guanosine triphosphate–binding protein RacI and reactive oxygen species (ROS) in inducing *MMP1* gene expression via the NF-κβ pathway following alterations of integrin mediated adhesion^34^. We noted a consistent upregulation of VTN, both at protein and mRNA level, in WJ-MSCs at 40 °C. Next we knocked down VTN expression using siRNA and studied its effect on the expression of the genes under study. There was an increase in *MMP1* gene expression. Interestingly, *p53 and p21* both showed a significant downregulation in mRNA expression, which further confirmed an inverse relation between p53/p21 and MMP1. Here we established a new link between VTN and p53, which to our knowledge, has not been shown before. Interestingly, p53 knock down did not affect VTN, suggesting that p53 might be placed downstream of VTN in the regulatory network. An earlier study on human endothelial cells had revealed that the ligation state of VTN receptor, integrin alphaV beta3, directly influenced p53 activity and p21 expression^35^.

A previous report suggested p21 to be one of the nodal points through which p53 inhibited cytokines and MMPs^20^. We noted a marked increase in both p53 and p21 in WJ-MSCs at 40 °C with a corresponding decrease in *MMP1*. On inhibiting p65 and p53, respectively, using siRNAs, p21 expression came down in both the cases and there was a simultaneous increase in *MMP1* expression. Moreover, as p21 is known to bind to and inhibit JNK activity, p21 might be suppressing MMP1 in WJ-MSCs at 40 °C via suppression of JNK pathway. In agreement, on inhibiting the JNK pathway at 40 °C, we observed a further suppression of *MMP1* expression. Increased levels of p53 and p21 were maintained. Overall, our study suggested a role of p53, acting via p21, in MMP1 suppression in WJ-MSCs at 40 °C, involving the JNK pathway.

**Figure 7:**
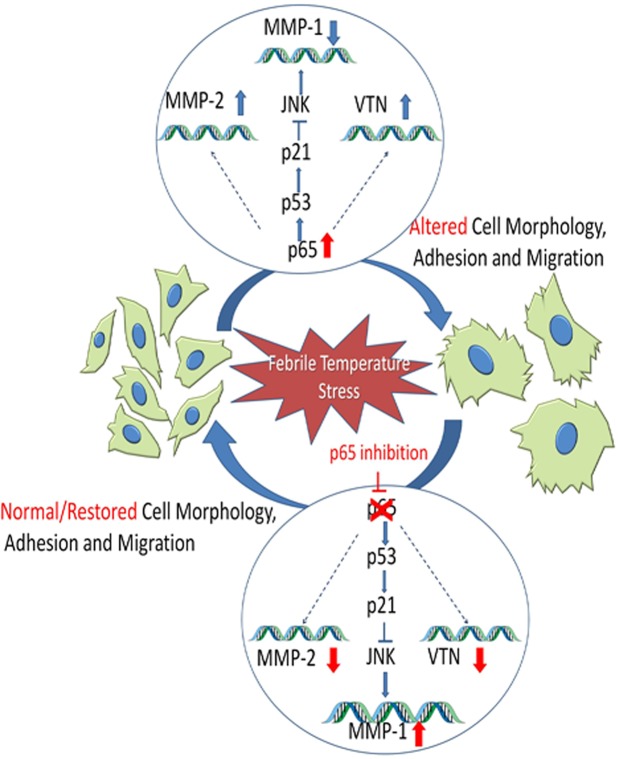
Temperature stress affects adhesion and migration of MSCs. Inflammation hallmark, febrile temperature stress led to alteration in cell morphology and fundamental properties of MSCs, such as adhesion and migration. Corresponding to these changes, expression of adhesion and migration related gene expression also got affected. Inhibition of NF-κß pathway resulted in reversal of altered physiological traits as well as gene expression of MMP-1 via p53/p21, acting through JNK pathway.

In conclusion, this is the first study to show an effect of elevated temperature stress on migration and adhesion of MSCs with an underlying involvement of the NF-κβ pathway, and the regulation of MMP1 by p53/p21 (Fig. 7). Our study provides evidence that VTN regulates p53 expression in MSCs under elevated temperature stress. Finally, based on our previous study and current findings, NF-κβ signaling emerges as the predominant pathway in controlling various processes of WJ-MSCs under febrile temperature stress.

## Methods

### Cell culture

Human umbilical cords were collected after full term births (cesarean or vaginal delivery) with informed consent from each donor and following the guidelines as approved by the Institutional Ethics Committee (IEC) and Institutional Committee for Stem Cell Research and Therapy (IC-SCRT) at IISER, Kolkata, India. WJ-MSCs were isolated from the umbilical cord by explant culture method as described earlier^36^.

WJ-MSCs were dissociated with TrypLE Express (Life Technologies) and cells were always plated at 5000 cells/cm^2^. TrypLE, being an animal origin-free recombinant enzyme and gentle on stem cells, was used in place of trypsin. WJ-MSCs between passages 4–6 were used for performing most of the experiments.

WJ-MSCs were first plated at 37 °C for 24 h. For 40 °C temperature treatment, this was followed by exposure to 40 °C for 48 h. For control condition, cells were continued to grow at 37 °C for the next 48 hs. For inhibitor experiments, respective vehicles or inhibitors were added after initial 24 h. PDTC and SP 600125 (both from Sigma-Aldrich, Saint Louis, USA) were added at 700 nM and 10 µM, respectively.

For siRNA transfections, WJ-MSCs were seeded onto 12-well plates and, at ~60% confluency, they are transfected with 30 nM each of p53 siRNA (Santa Cruz Biotechnology, Inc., CA, USA) and esiRNA for p65 and VTN along with corresponding MISSION® siRNA Universal Negative Control (NC siRNA) (all three from Sigma-Aldrich). The transfections were performed using Lipofectamine 3000 in opti MEM medium (both from Life Technologies) as per the manufacturer’s protocol. After 8 h of transfection, the cultures were exposed to 40 °C temperature stress for the next 36–40 h, followed by lysate preparation.

### Cell Spread Area

To assess the change in morphology, in terms of cell spread area under the different experimental conditions, phase contrast images of WJ-MSCs were captured using Olympus IX81 (Olympus, Tokyo, Japan) inverted microscope via micromanager software. For calculation of cell spread area using ImageJ (NIH, USA) software, the cell boundaries were marked using free-hand selection tool and area was measured using measure tool in analyze tool bar. From each biological sample, cell spread area of 50 cells was calculated and the data was plotted by fitting to Gaussian equation using MATLAB 2009 software to get the cell spread area distribution across the population of cells.

### Wound Healing assay

To study the effect of 40 °C temperature stress on migration of WJ-MSCs *in vitro*, confluent monolayer cultures were scraped with a pipette tip to make scratches of uniform width. Phase contrast images were captured at an interval of 2 h till wound closure using Olympus IX81 (Olymus) inverted microscope. The migration of WJ-MSCs was quantified in terms of wound closure rate by measuring the width of the wound across the time points using ImageJ software. In brief, wound width was measured by calculating average length of ~20 equally spaced lines drawn using straight line tool, covering the wound horizontally for the different time points.

### Time Lapse Imaging of cell migration

Cell migration was monitored by live cell Differential Interface Contrast (DIC) time lapse imaging. WJ-MSCs were seeded at a density of 4000 cells/cm^2^ in 35 mm confocal glass bottom dishes (SPL Lifesciences) and exposed to 40 °C temperature treatment as mentioned earlier. The DIC time lapse videos were recorded over a period of 3 hours by capturing live cell images at a frequency of one frame per minute in confocal microscope LSM 710 (Axio Observer Z1, Carl Zeiss, Germany) equipped with an environmental chamber set at 5% CO_2_ and 37° or 40 °C, based on the experiment.

Average migration speed, calculated as micrometers per hour (µm/h), was determined for individual cells by tracking the total distance covered by the center of a cell using the ZEN 2010 software (Zeiss) using ZEN 2010 software (Zeiss). Thirty cells per sample and three different biological samples were used.Cell trajectory pattern, MSD and directionality ratio were calculated using DiPer software^10^. The xy co-ordinates of cells were obtained using ImageJ software. In brief, cells were marked, considering nucleus to be the reference point for each of the cells, using the multi-point selection tool and the XY co-ordinates were obtained using measure tool option in analyze tool bar of the ImageJ software.

### De-adhesion dynamics

To understand the effect of febrile temperature on WJ-MSC’s de-adhesion dynamics, time lapse imaging was done for the live cells in the presence of TrypLE Express Enzyme (Life Technologies). Live cell imaging was performed in Olympus IX83 with ORCA Flash 4.0 (Hamamatsu) camera via cellSens imaging software, equipped with temperature controlled chamber which helped to maintain 37 °C temperature during the experiment. After removal of medium and DPBS (Thermo Fisher Scientific) wash, 500 µl of 1:2 dilution of TrypLE in DPBS was added. Images were taken at 10x magnification at an interval of 10 s for a period of 500 s as the cells rounded off. Cell spread area of 15 individual cells from each of the three independent biological samples were measured across the time period till the cells rounded off and detached from the surface, using ImageJ (NIH) software. The normalized cell area was plotted against time and was fitted in Boltzmann’s equation using GraphPad Prism 5 (GraphPad, La Jolla, CA, USA) software and the two time constants, τ1 and τ2, were obtained from the graph which quantified the de-adhesion dynamics of the cells^24^.

### Complementary DNA synthesis and human Extracellular Matrix and Adhesion Molecule RT^2^ Profiler PCR Array

Total RNA was isolated from three different sets of biological samples of WJ-MSCs, each set comprising of 40 °C temperature treated and its respective control condition WJ-MSCs. RNA was isolated using RNAeasy Plus Mini kit (Qiagen, Hilden, Germany) as per the manufacturer’s protocol and RNA yield was quantified using Nanodrop 1000 spectrophotometer (Thermo Scientific, Asheville, NC, USA). An equal amount of RNA, pooled from three different samples, was used for cDNA synthesis for each of the 40 °C treated and control condition, respectively, using RT^2^ First strand kit (Qiagen). cDNA was mixed with 2x RT^2^ SYBR Green ROX qPCR Mastermix (Qiagen) and 25ul of the mixture was added to each well of Human Extracellular Matrix and Adhesion Molecule RT^2^ Profiler PCR Array (Qiagen) plate. Array run was performed in ABI Biosystems StepOnePlus instrument (Applied Biosystems, Carlsbad, CA, USA) and the data was analysed using Step-onePlus version v.2.2 software (Applied Biosystems). A cycle threshold of ≤35 was considered for calculating the fold change in gene expression and the arithmetic mean of cycle threshold values of the 5 different house-keeping genes was used for normalizing the data.

### Validation of PCR Array data via RT-PCR

To validate the gene expression pattern of the ECM and Adhesion molecule PCR array, semi-quantitative RT-PCR analysis was performed for a few randomly selected genes from the PCR array. cDNA was prepared using High-Capacity cDNA Reverse Transcription Kit (Applied Biosystems). Semi-quantitative RT-PCR was performed using Taq DNA polymerase kit (Invitrogen, Carlsbad, CA, USA) for each of the individual sample sets. GAPDH was used as an internal control. The primer sequences used in RT-PCR analysis with the respective amplicon sizes are listed in Supplementary Table 2.

### RNA isolation and cDNA synthesis

Total RNA was isolated using Tri-Reagent (Sigma-Aldrich) and the RNA yield was quantified using Nanodrop 1000 spectrophotometer (Thermo Scientific). 250–700 ng of RNA was used for cDNA synthesis using High-Capacity cDNA Reverse Transcription Kit (Applied Biosystems) or Verso cDNA Synthesis kit (Thermo Scientific).

### Quantitative reverse transcription polymerase chain reaction

qRT-PCR reactions were done with PowerUp SYBR^TM^ Green Master mix (Applied Biosystems) using ABI Biosystems Step-onePlus instrument (Applied Biosystems). StepOnePlus software (version v.2.2; Applied Biosystems) was used to analyze data using the 2^−ΔΔCT^ method. Melting curve analysis was done to confirm single, specific PCR products. GAPDH was considered as the endogenous control gene. The primer sequences and amplicon sizes are listed in Supplementary Table 2.

### Western Blot

Whole cell lysates were prepared on ice in RIPA lysis buffer containing protease inhibitor cocktail (Sigma-Aldrich). Polyacrylamide gel electrophoresis and Western blotting were carried out as per standard protocols. Primary and secondary antibodies used were anti-p21 (Santa Cruz Biotechnology Cat# sc-271532, RRID:AB_10650266), anti-p53 (Santa Cruz Biotechnology Cat# sc-126, RRID:AB_628082), anti-p65 (Santa Cruz Biotechnology Cat# sc-8008, RRID:AB_628017), anti-GAPDH (Santa Cruz Biotechnology Cat# sc-47724, RRID:AB_627678) and HRP-linked anti-mouse IgG (Cell Signaling Technology, Inc., Denvers, MA, USA, Cat# 7076, RRID:AB_330924). The immunoreactivity was detected using ECL-Plus kit (G-Biosciences, Maryland Heights, MO, USA) and the blots were imaged using G: Box (Syngene, Frederick, MD, USA).

### Statistical Analysis

All data are presented as mean ±standard error of the mean from at least 3 independent biological samples. Data analysis and graphical representations were performed using GraphPad Prism 5 and 8 softwares (GraphPad). Statistical comparisons were assessed using the two-tailed Student t-test or one-way ANOVA. Significance was accepted at P ≤ 0.05.

## Supplementary information

Supplementary video 1.

Supplementary video 2.

Supplementary file.

## Data Availability

The data that support the findings of this study are available on request from the corresponding author.
