# Supplementary material for: Altered Adhesion and Migration of Human Mesenchymal Stromal Cells under Febrile Temperature Stress Involves NF-κβ Pathway
Source: Sci Rep. 2020 Mar 11;10:4473. doi: 10.1038/s41598-020-61361-z (PMC7066177; doi:10.1038/s41598-020-61361-z)
Supplement: Supplementary file 3 — Supplementary file. [file 41598_2020_61361_MOESM3_ESM.doc]

**Altered Adhesion and Migration of Human Mesenchymal Stromal Cells under Febrile Temperature Stress Involves NF-κβ Pathway**

Ankita Sen and Malancha Ta.

Indian Institute of Science Education and Research, Kolkata, India.

Ankita Sen, Email: as17rs015@iiserkol.ac.in

Malancha Ta, Email: malancha.ta@iiserkol.ac.in

**Running title:**  Role of NF-κβ in adhesion and migration of MSCs

**Corresponding Address:**

Malancha Ta, PhD,

Lab 227, Research Complex

Indian Institute of Science Education and Research (IISER)-Kolkata,

Mohanpur Campus, Dist: Nadia, West Bengal-741246, India.

Phone: 033-61360000 Extn 1217

Email: malancha.ta@iiserkol.ac.in

| HUMAN EXTRACELLULAR MATRIX AND ADHESION MOLECULE GENES SCREENED USING THE RT^2^ PROFILER™ PCR ARRAY | | | | | | | | | | | |
| --- | --- | --- | --- | --- | --- | --- | --- | --- | --- | --- | --- |
| *ADAMTS1* | *ADAMTS13* | *ADAMTS8* | *CD44* | *CDH1* | *CLEC3B* | *CNTN1* | *COL11A1* | *COL12A1* | *COL14A1* | *COL15A1* | *COL16A1* |
| *COL1A1* | *COL4A2* | *COL5A1* | *COL6A1* | *COL6A2* | *COL7A1* | *COL8A1* | *CTGF* | *CTNNA1* | *CTNNB1* | *CTNND1* | *CTNND2* |
| *ECM1* | *FN1* | *HAS1* | *ICAM1* | *ITGA1* | *ITGA2* | *ITGA3* | *ITGA4* | *ITGA5* | *ITGA6* | *ITGA7* | *ITGA8* |
| *ITGAL* | *ITGAM* | *ITGAV* | *ITGB1* | *ITGB2* | *ITGB3* | *ITGB4* | *ITGB5* | *ANOS1* | *LAMA1* | *LAMA2* | *LAMA3* |
| *LAMB1* | *LAMB3* | *LAMC1* | *MMP1* | *MMP10* | *MMP11* | *MMP12* | *MMP13* | *MMP14* | *MMP15* | *MMP16* | *MMP2* |
| *MMP3* | *MMP7* | *MMP8* | *MMP9* | *NCAM1* | *PECAM1* | *SELE* | *SELL* | *SELP* | *SGCE* | *SPARC* | *SPG7* |
| *SPP1* | *TGFB1* | *THBS1* | *THBS2* | *THBS3* | *TIMP1* | *TIMP2* | *TIMP3* | *TNC* | *VCAM1* | *VCAN* | *VTN* |
| *ACTB^a^* | *B2M^a^* | *GAPDH^a^* | *HPRT1^a^* | *RPLP0^a^* | *HGDC^b^* | *RTC^c^* | *RTC^c^* | *RTC^c^* | *PPC^d^* | *PPC^d^* | *PPC^d^* |

Supplementary Table 1: The panel of 84 genes, defining cell-cell and cell-matrix interaction in Human Extracellular Matrix and Adhesion Molecule Genes RT^2^ profiler™ PCR array

^a^Housekeeping genes.

^b^Human genomic DNA contamination control.

^c^Reverse transcription control.

^d^Positive polymerase chain reaction control

Supplementary Table 2: Primer sequences used for quantitative and semi-quantitative Reverse Transcriptase Polymerase

Chain Reaction (RT-PCR).

| Gene | Forward (5’-3’) | Reverse (5’-3’) | Amplicon(bp) |
| --- | --- | --- | --- |
| *COL1A1* | GGACACAATGGATTGCAAGG | TAACCACTGCTCCACTCTGG | 460 |
| *COL11A1* | AGCACAGACGGAGGCAAAC | AGAAGATCAGAATCCCTGCCG | 234 |
| *COL12A1* | ACCCACCTTCCGACTTGAATT | TAGGCCCATCTGTTGTAGGG | 122 |
| *C0L14A1* | AAGCCCAGAGTCAAAGTTGTGGA | CCATGAACCATCGACCAGGA | 123 |
| *GAPDH* | GAGTCAACGGATTTGGTCGT | TTGATTTTGGAGGGATCTCG | 248 |
| *ITGB1* | GTTACACGGCTGCTGGTGTT | CTACTGCTGACTTAGGGATC | 264 |
| *MMP1* | CCAGTATGCACAGCTTTCCTC | TGCCTCCCATCATTCTTCAGG | 154 |
| *MMP2* | ACATCAAGGGCATTCAGGAGC | CACAGTCCGCCAAATGAACC | 182 |
| *MMP2* | GGCCCCTGTCACTCCTTGAGAT | GGCATCCAGGTTATCGGGGA | 474 |
| *CDKN1A* | GAGGCCGGGATGAGTTGGGAGGAG | CAGCCGGCGTTTGGAGTGGTAGAA | 220 |
| *TP53* | GAGCTGAATGAGGCCTTGGA | CTGAGTCAGGCCCTTCTGTCTT | 151 |
| *VTN* | GGGTCTACTTCTTCAAGGGGAA | AATGAACTGGGGCTGTCTGG | 197 |


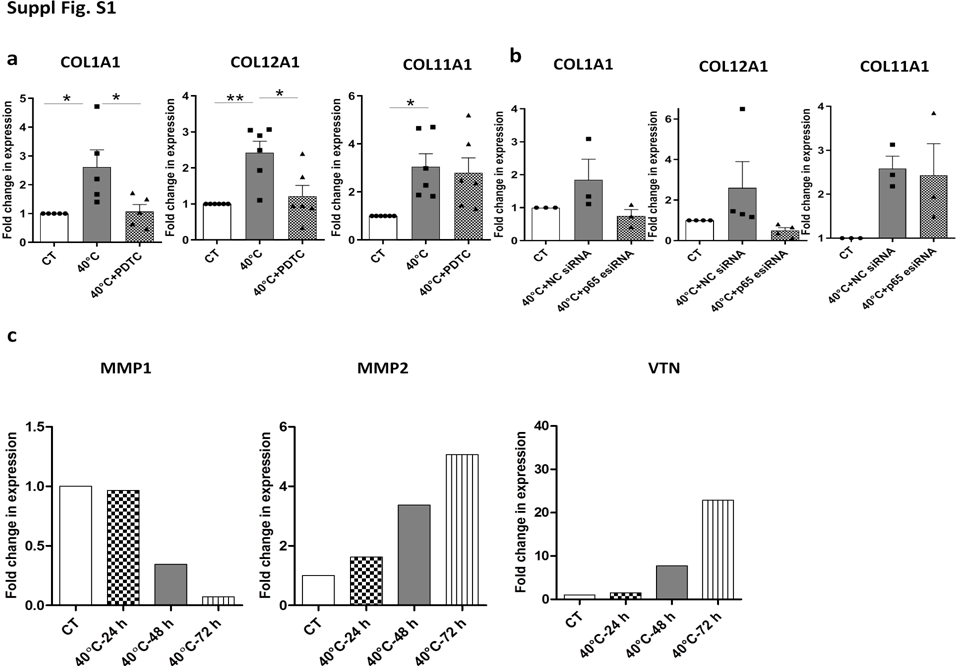


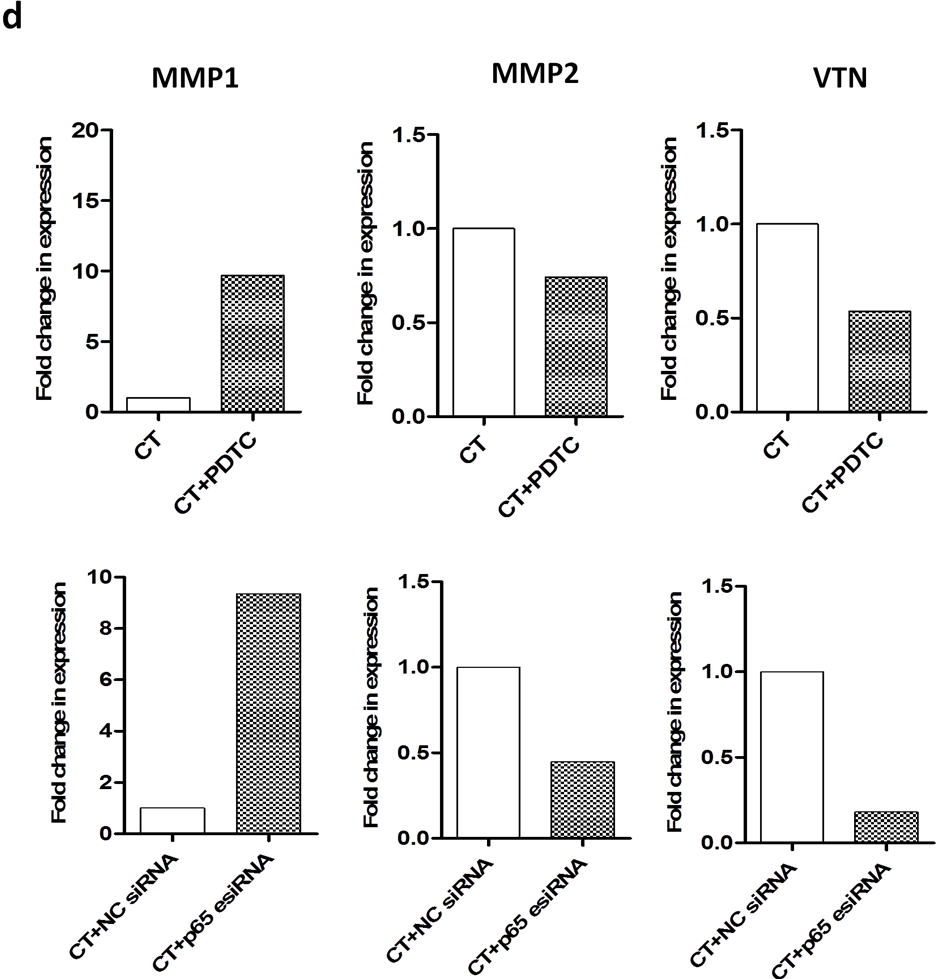


**Supplementary Figure S1**

Effect of NF-κβ inhibition on *COL* family of genes. WJ-MSCs were treated without and with 700 nM PDTC at 40ºC. qRT-PCR detection of *COL1A1, COL12A1* and *COL11A1* is reported. *represents p<0.05, n=3 (a). WJ-MSCs were transfected with a NC siRNA or a *p65*-targeted esiRNA for 48 h at 40ºC. qRT-PCR detection of *COL1A, COL12A1* and *COL11A1* is shown, n=3 (b) WJ-MSCs were exposed to 40ºC for the indicated time periods. qRT-PCR detection and comparison at the different time points for the genes *MMP1, MMP2* and *VTN* are displayed, n=2 (c). WJ-MSCs treated with 700 nM PDTC at 37ºC. qRT-PCR detection of *MMP1, MMP2* and *VTN* is shown, n=2. WJ-MSCs were transfected with a NC siRNA or a *p65*-targeted esiRNA for 48 h at 37ºC. qRT-PCR detection of *MMP1, MMP2* and *VTN* is shown, n=1 (d).Values are expressed as means± SEM and *GAPDH* was used as an endogenous control.


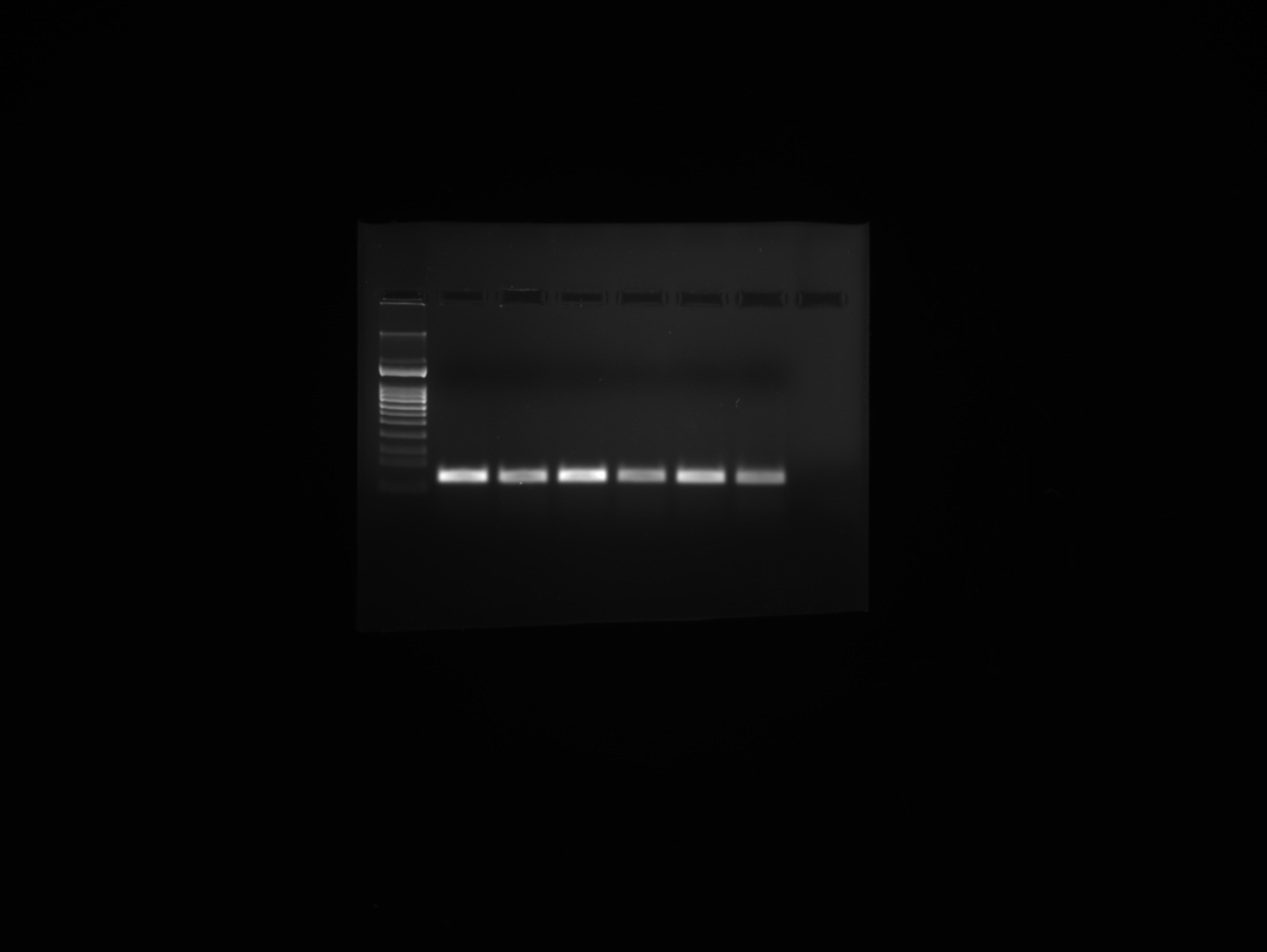

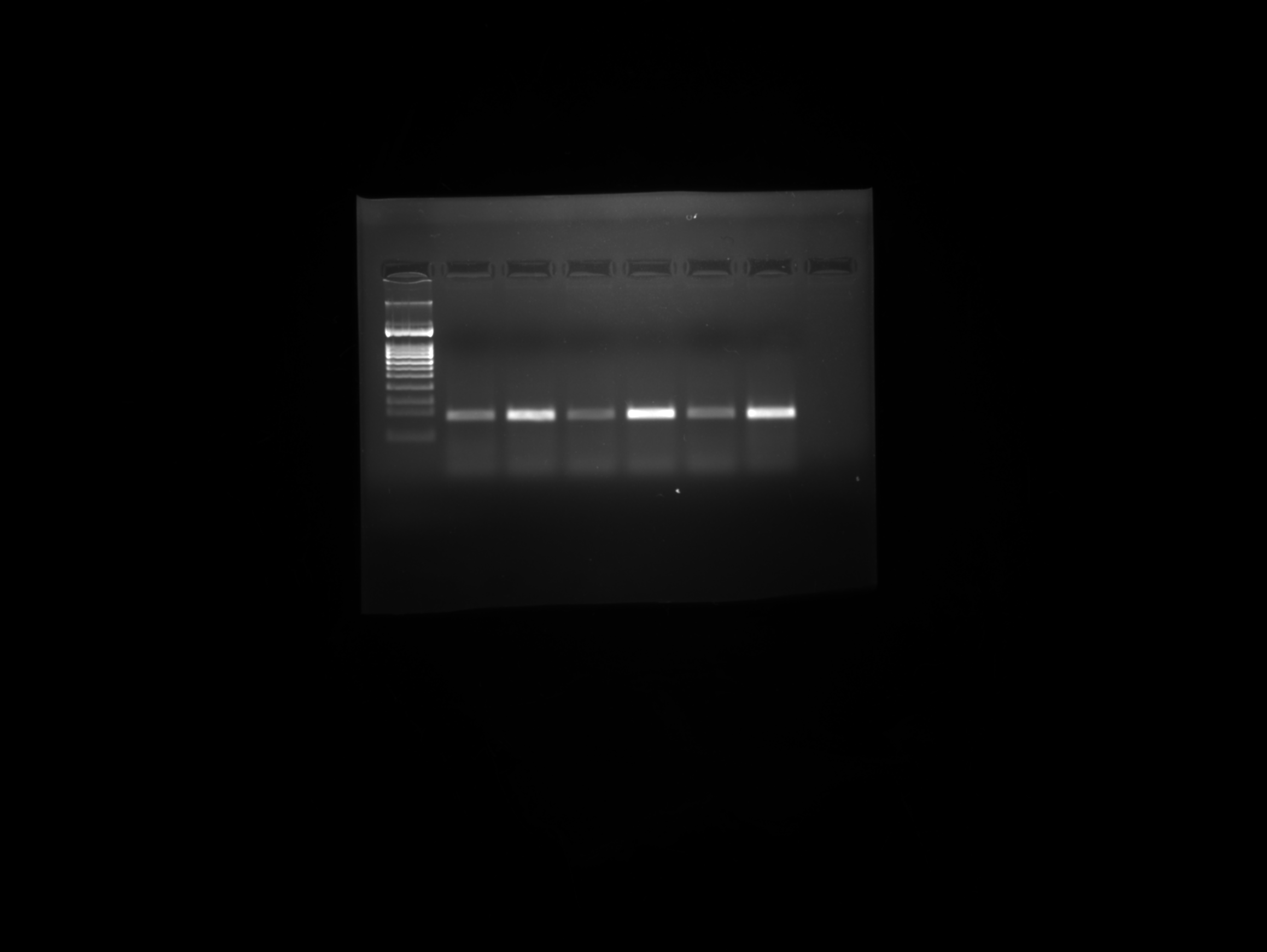

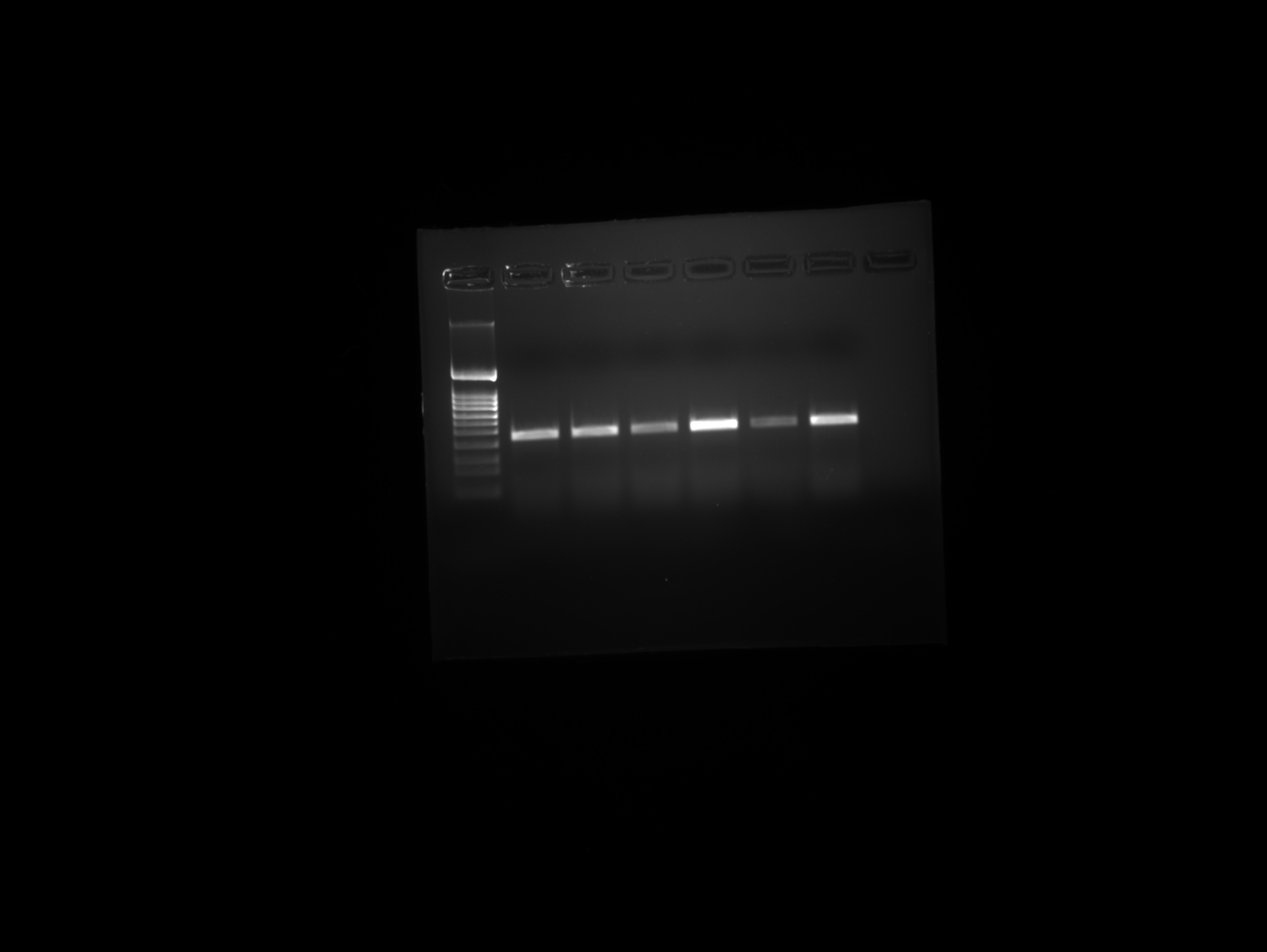


**
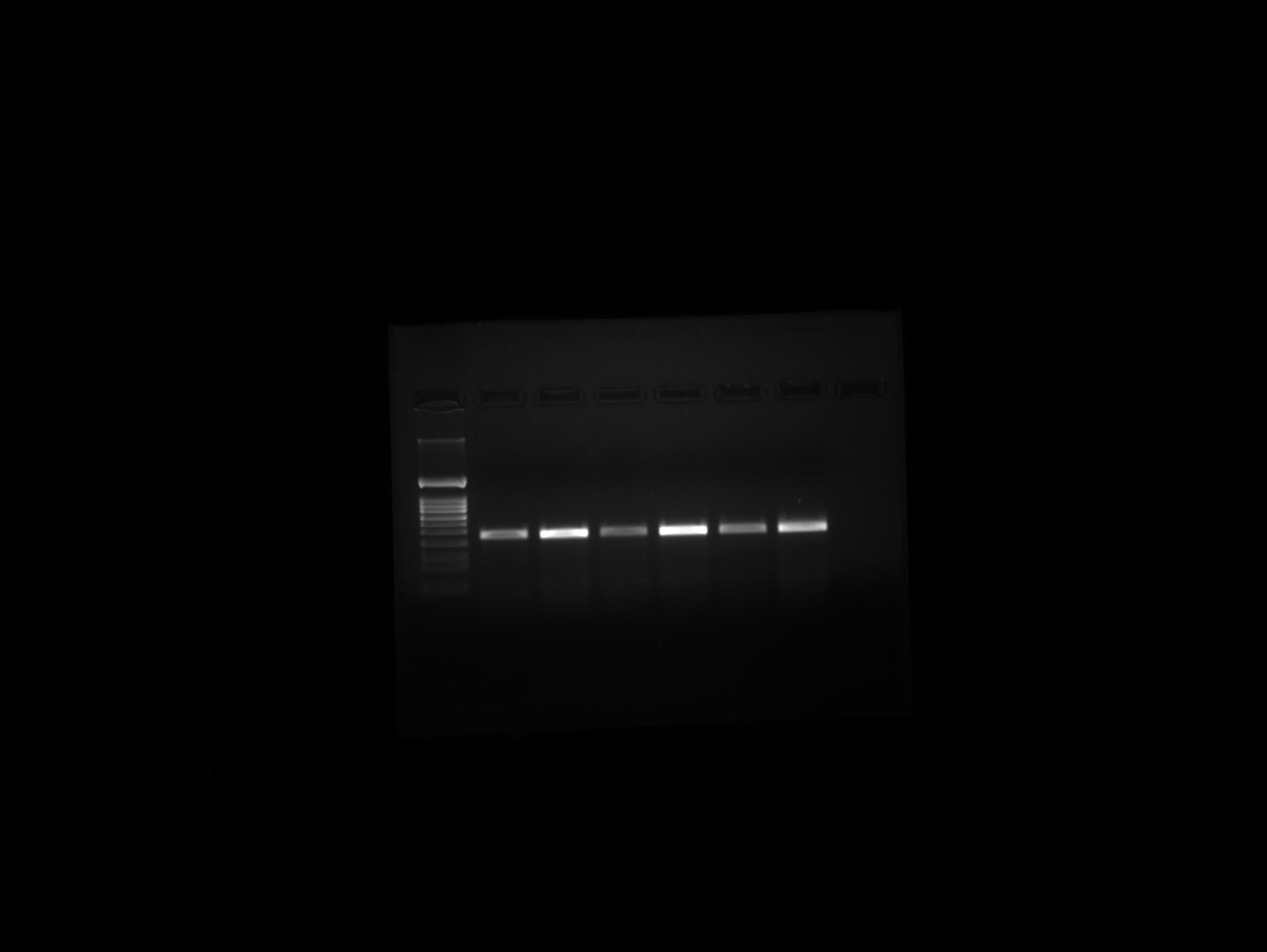
**

**a**

**b**

**c**

**e**

**g**

**d**


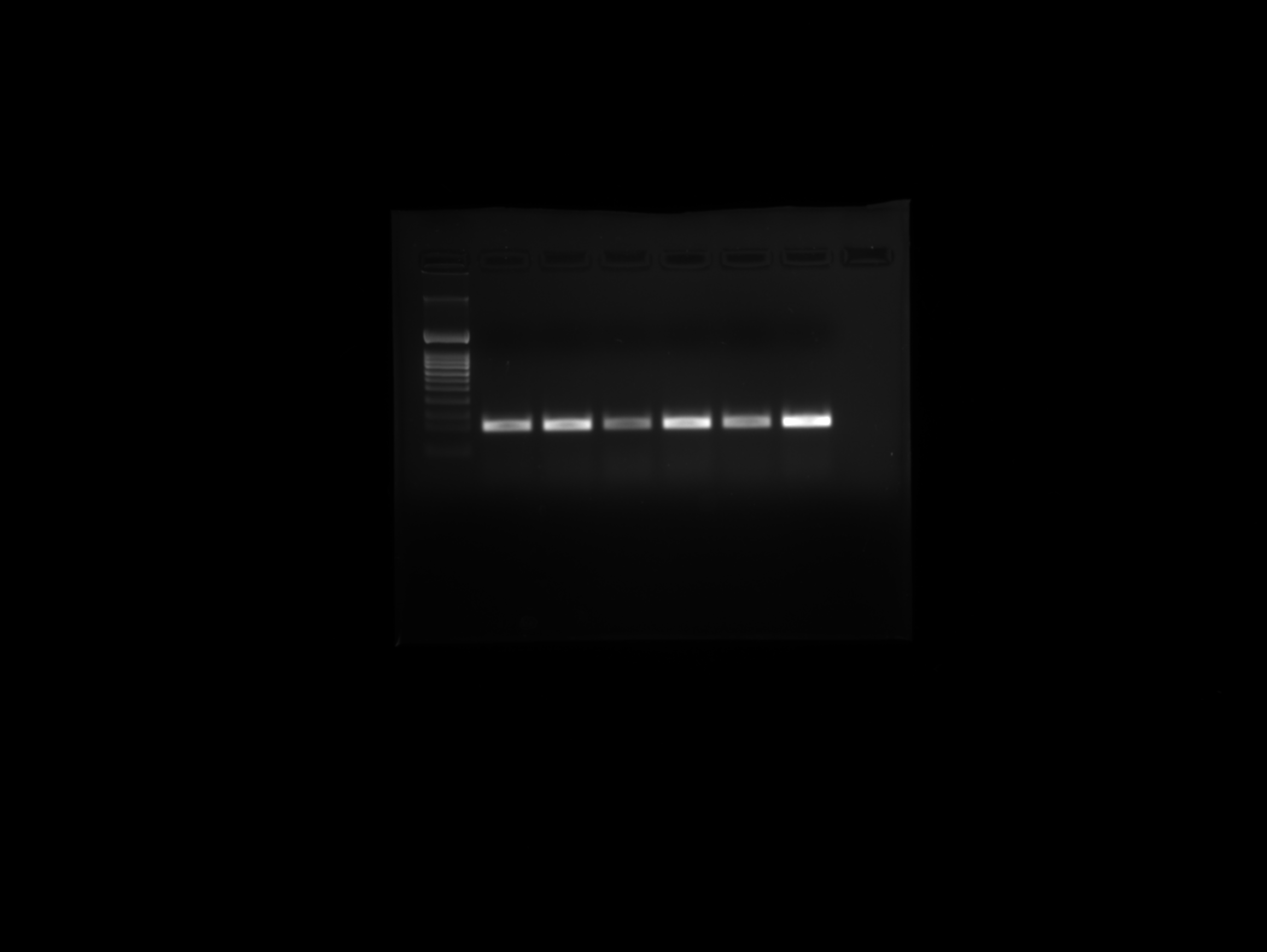

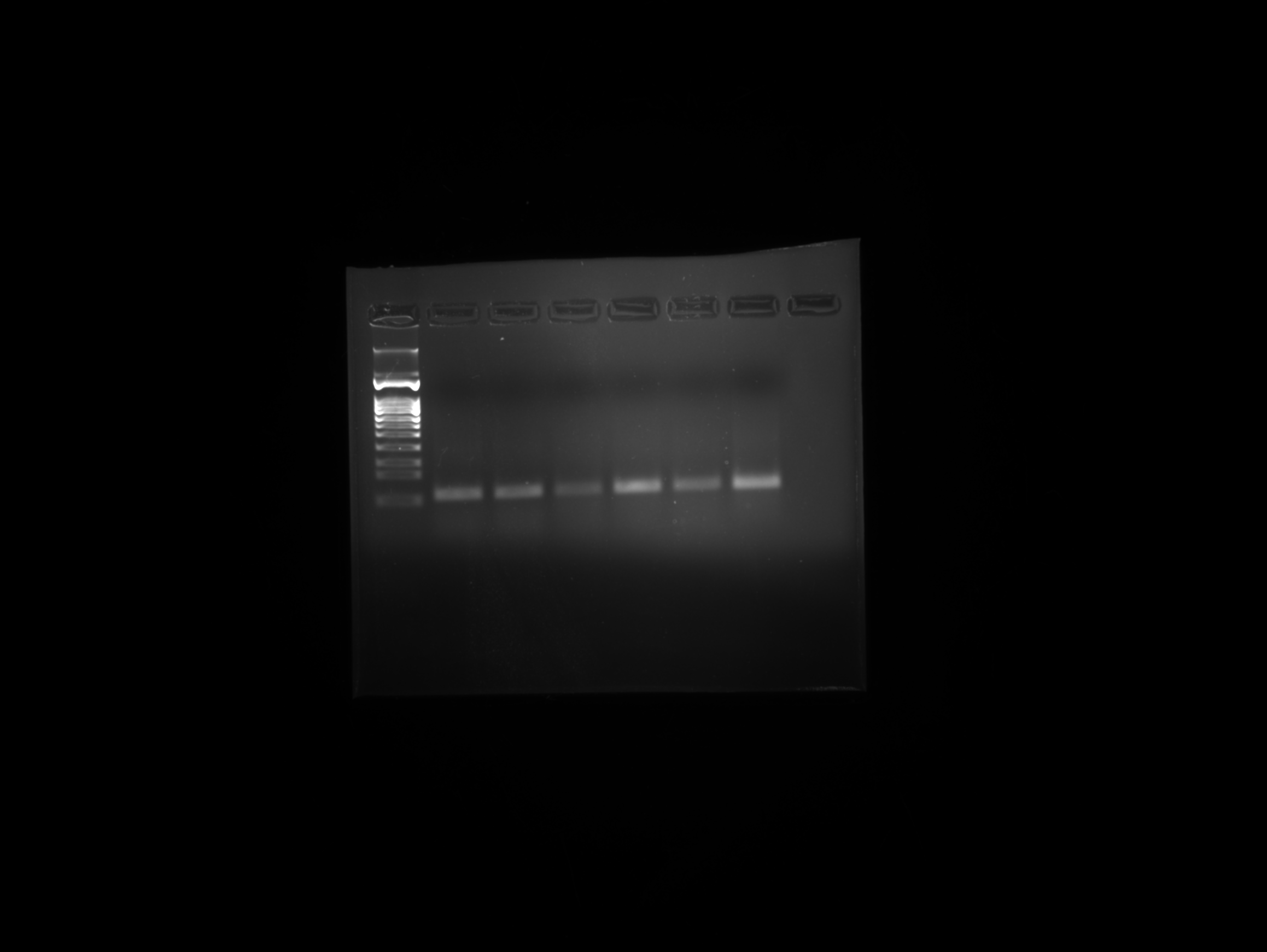

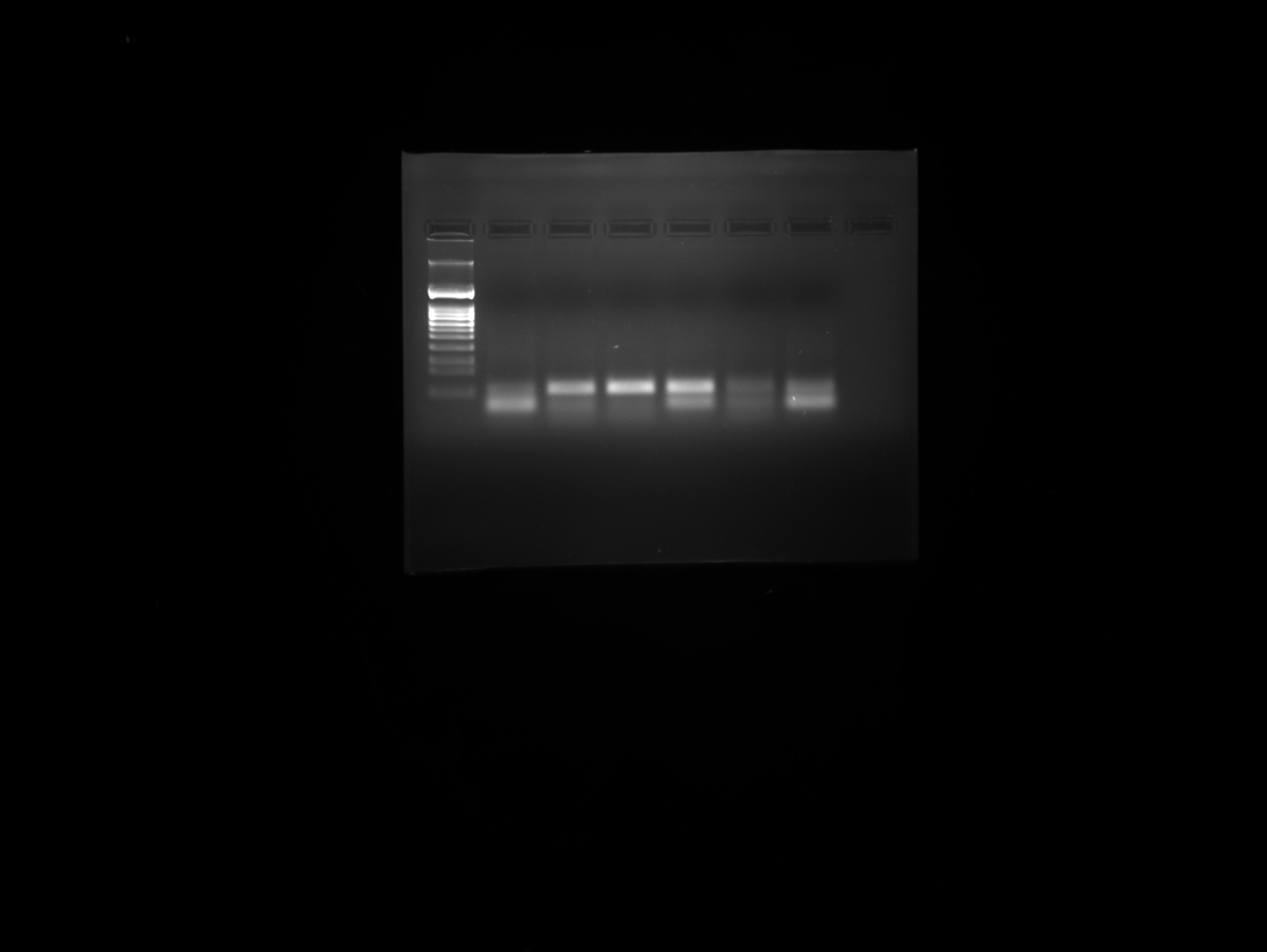


**f**


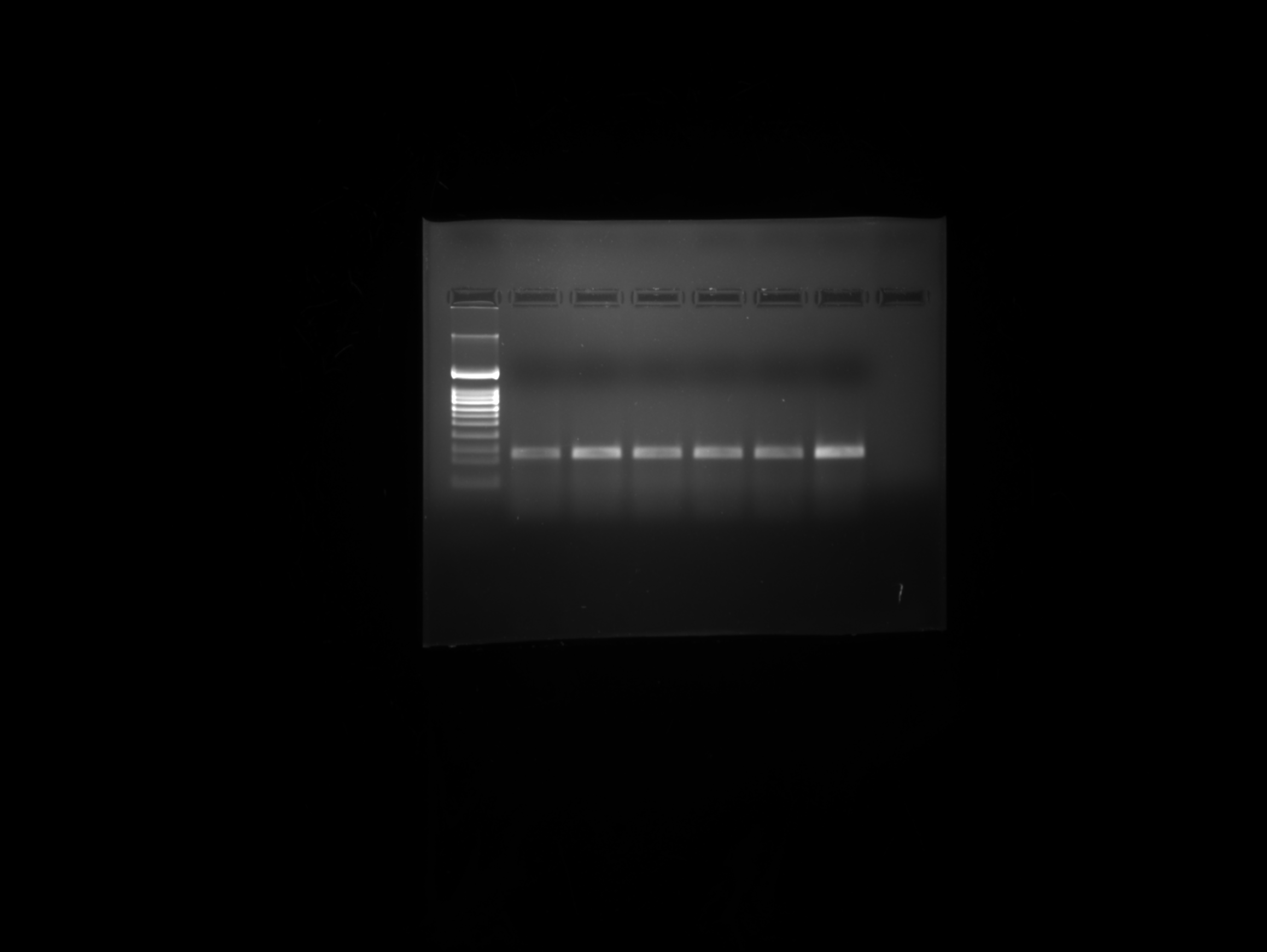

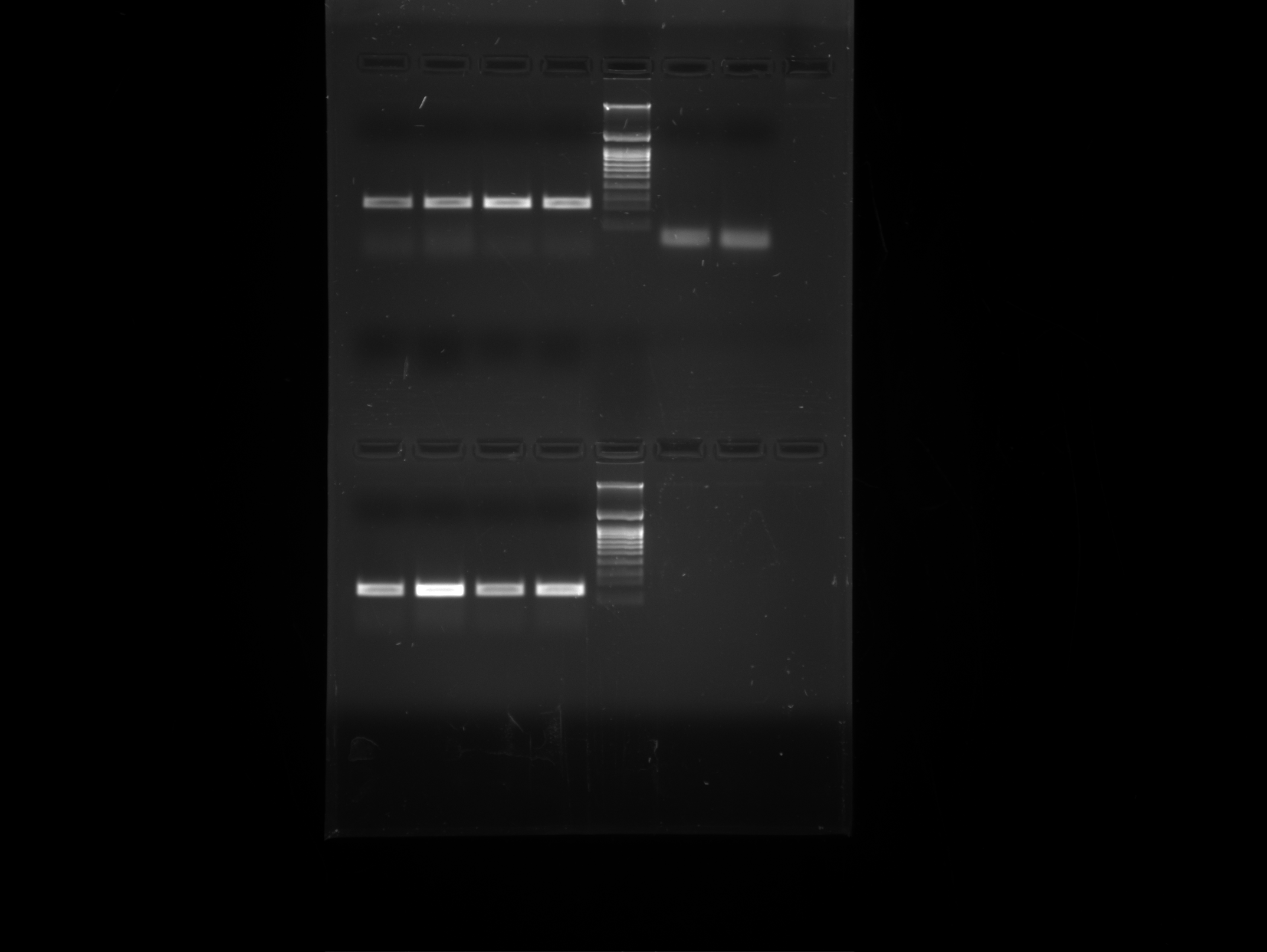

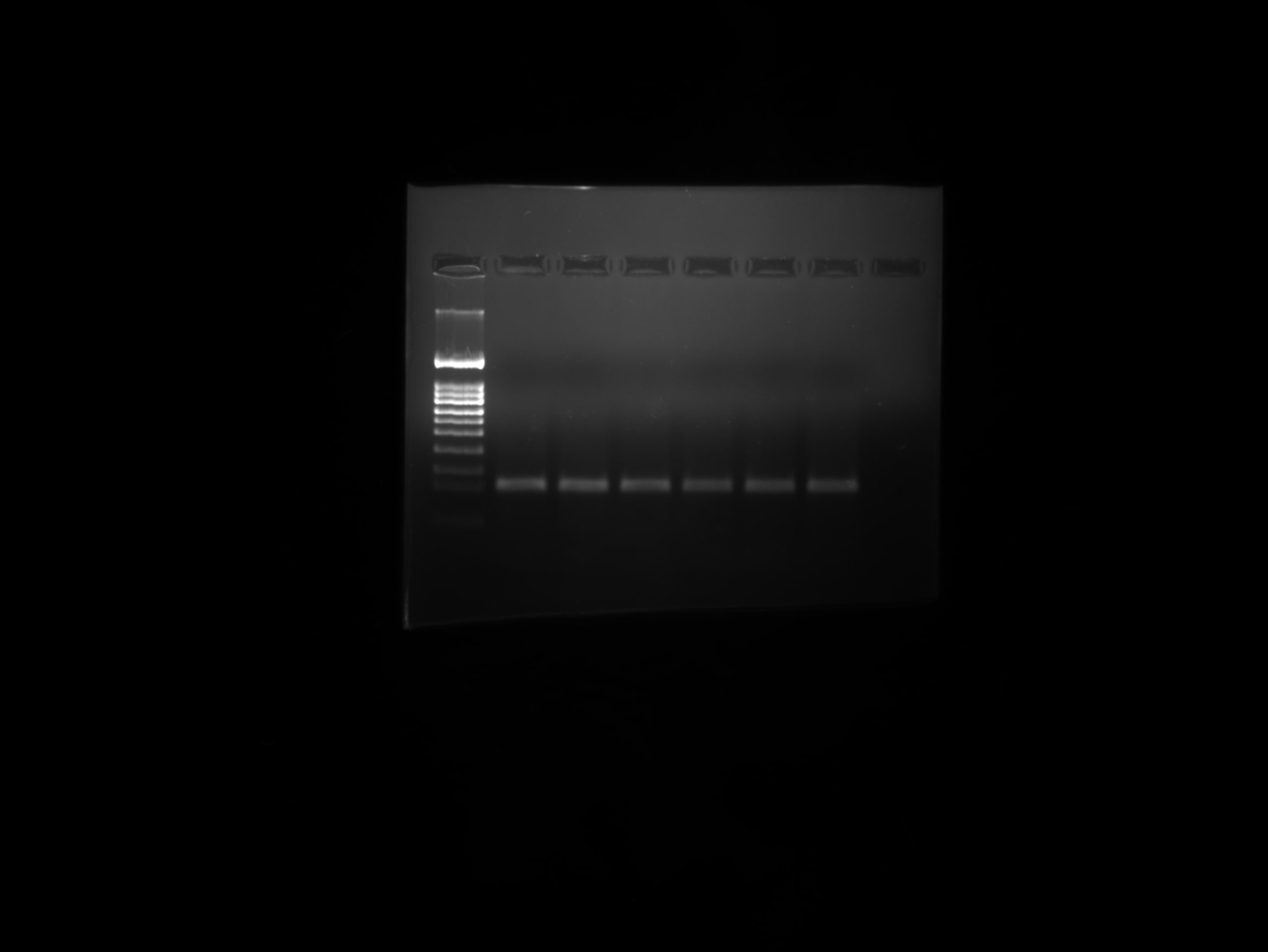

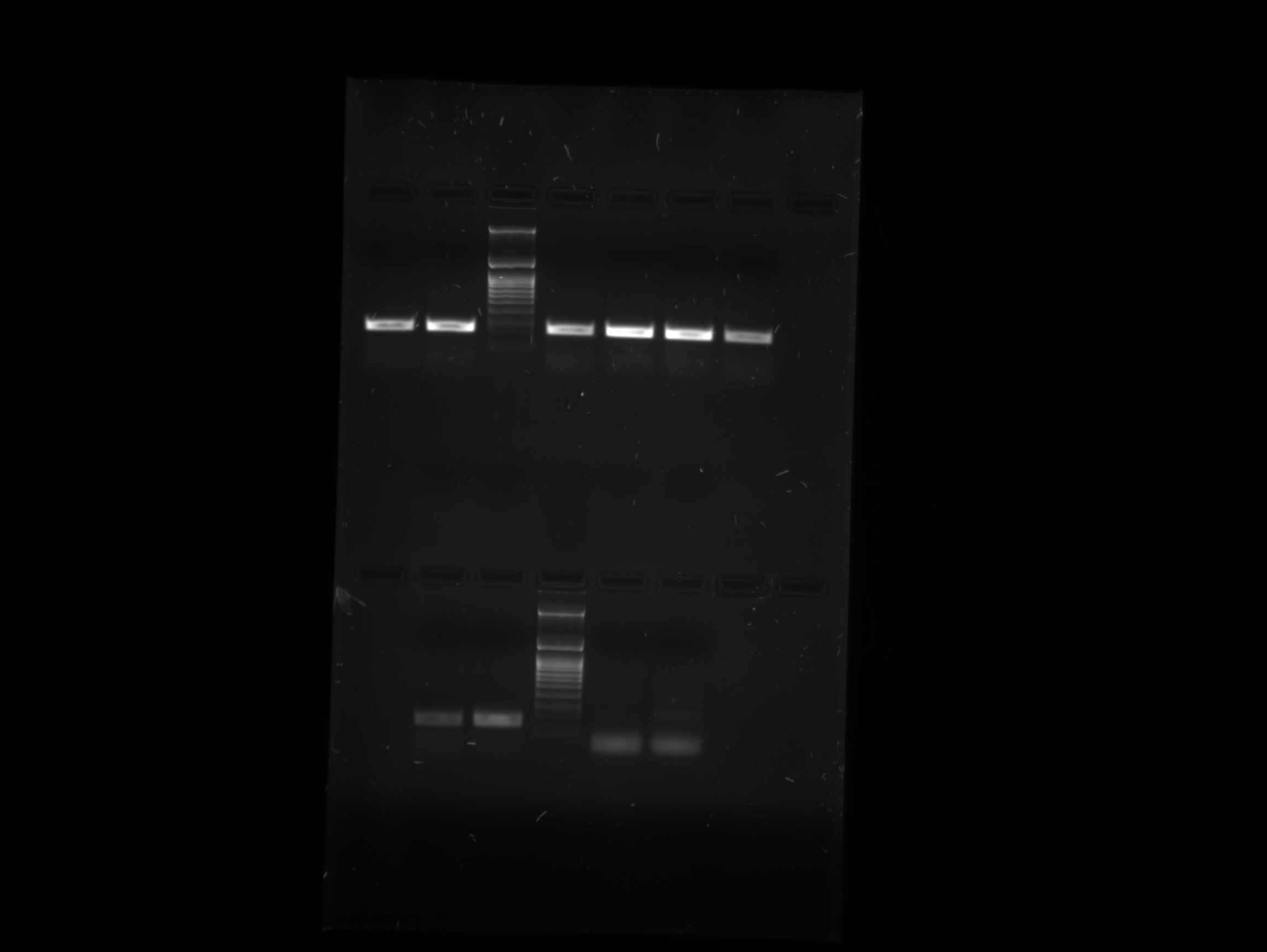


**h**

**i**

**j**

**k**

Supplementary Figure S2: Original agarose gel electrophoresis images of Figure 2g . Following are the genes which are represented in this figure. *MMP1* (a), *MMP2* (b), *VTN* (c), *COL1A1* (d), *COL11A1* (e), *COL12A1* (f), *COL14A1* (g), *TGFB1*(h), *TIMP3* (i,j) and *GAPDH* (k).


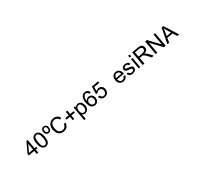

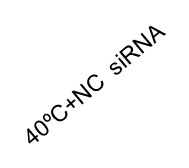

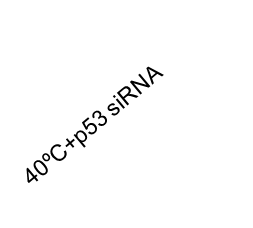


**Supplementary Figure S3:**


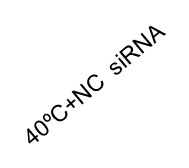


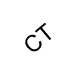

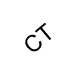


a

b


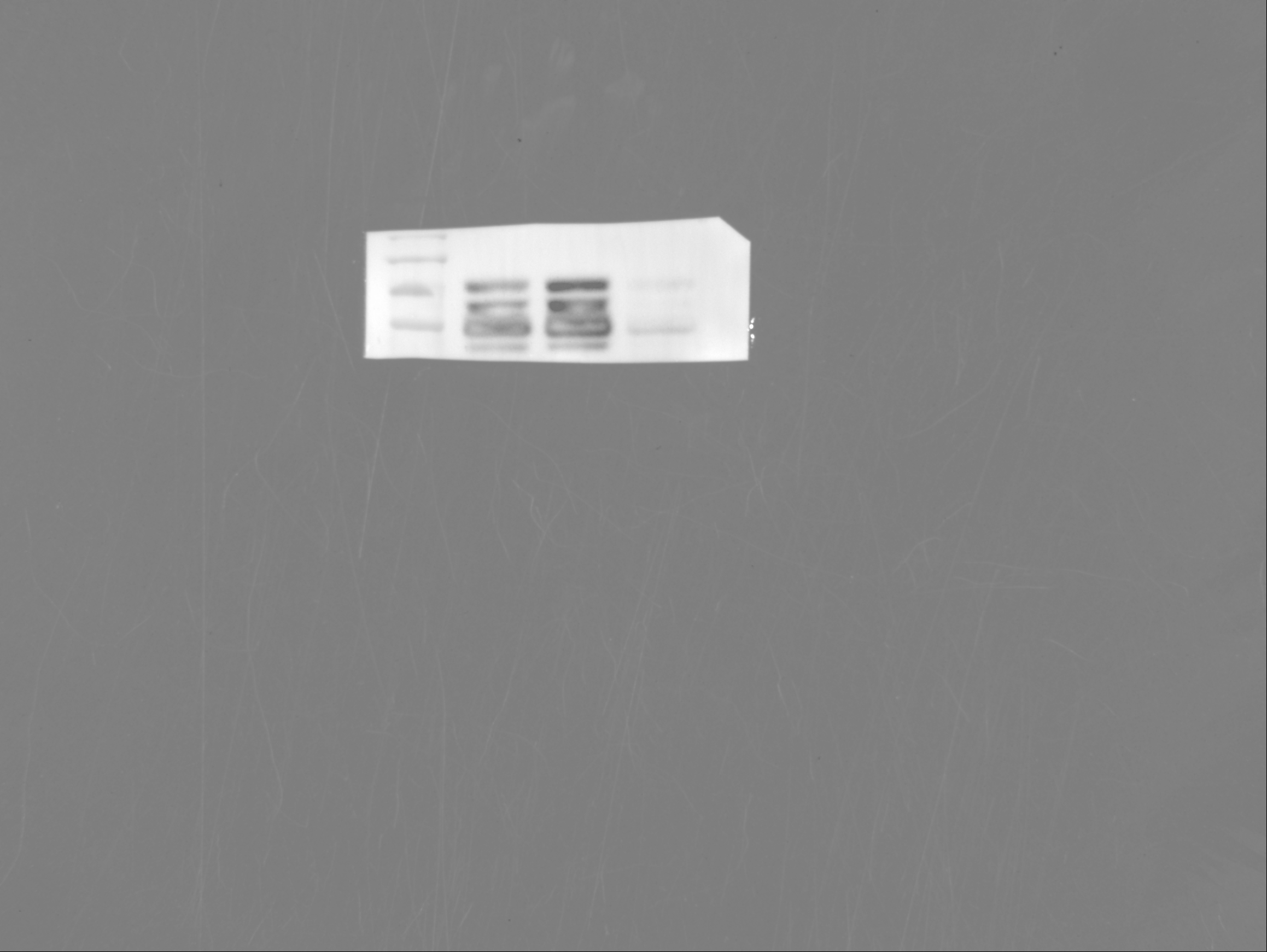

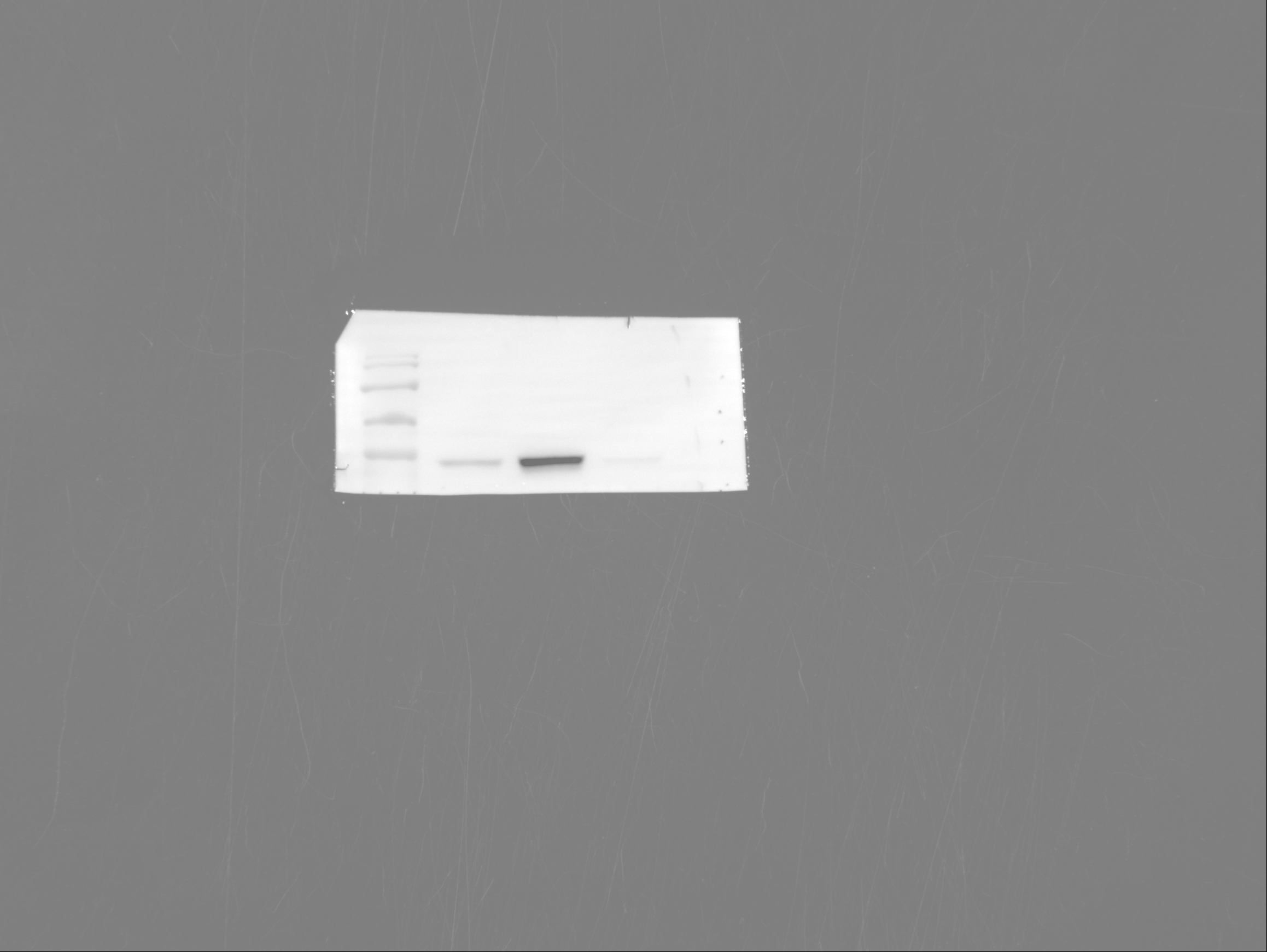


P65

P53


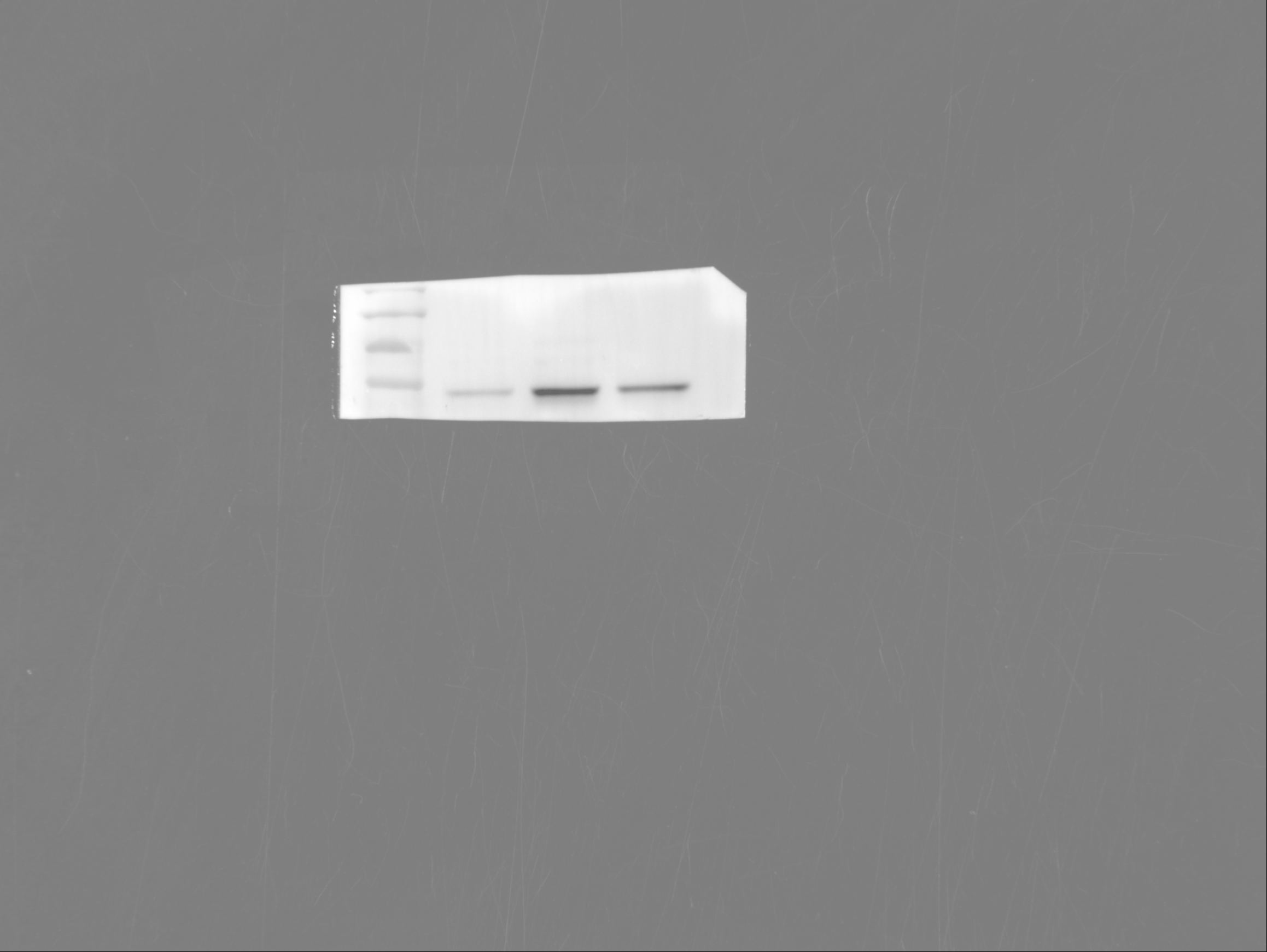

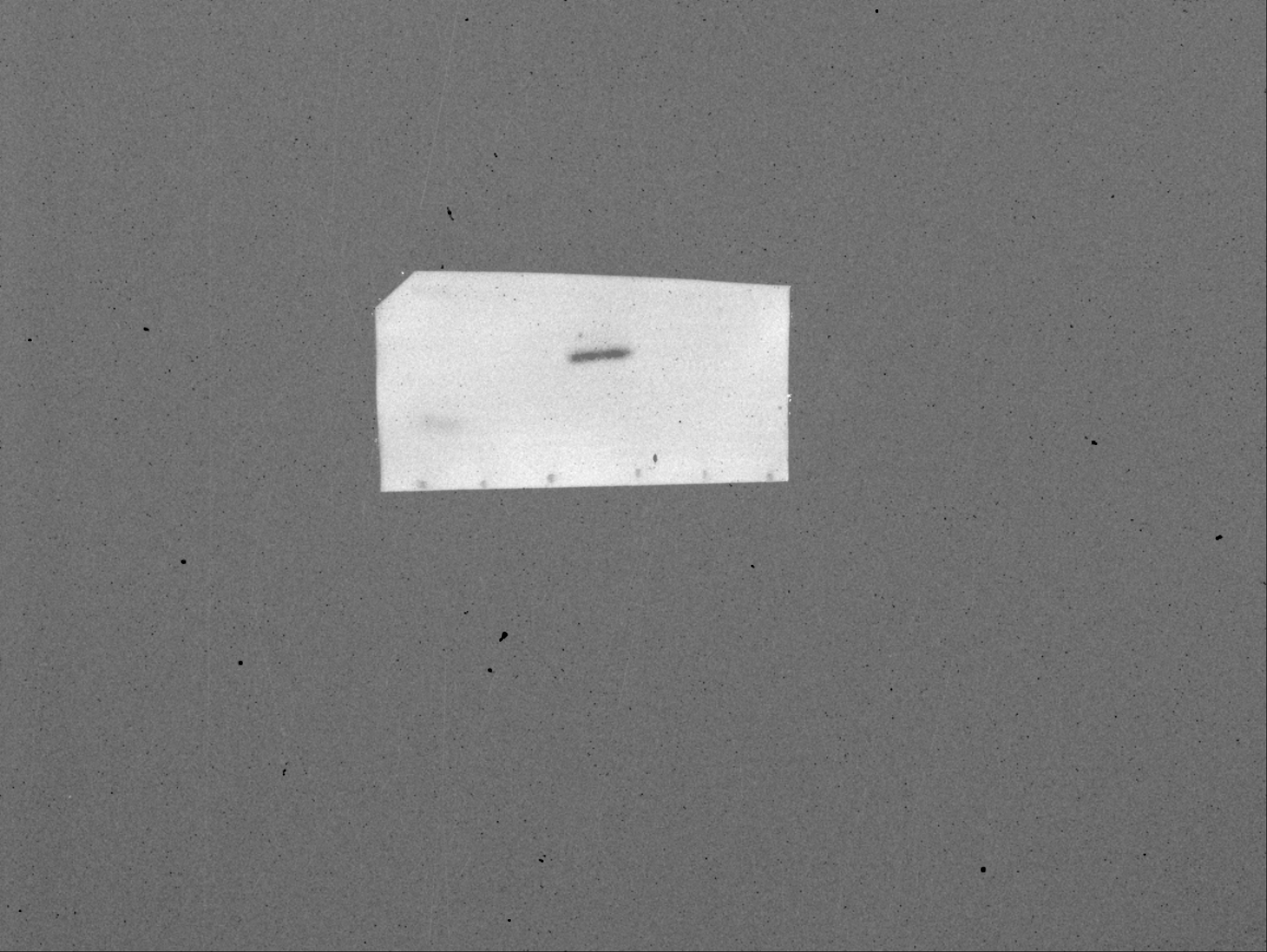


P53

P21


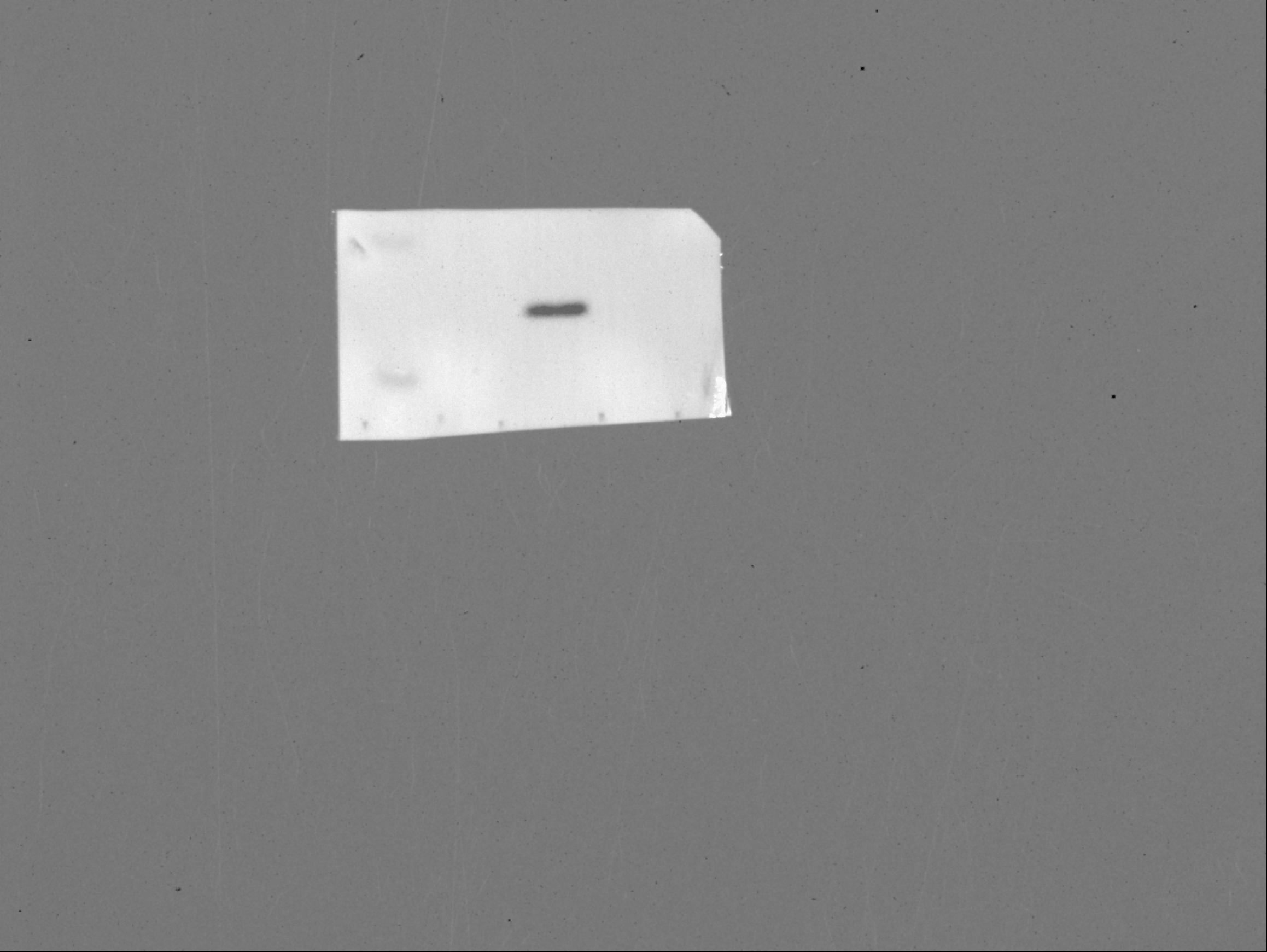

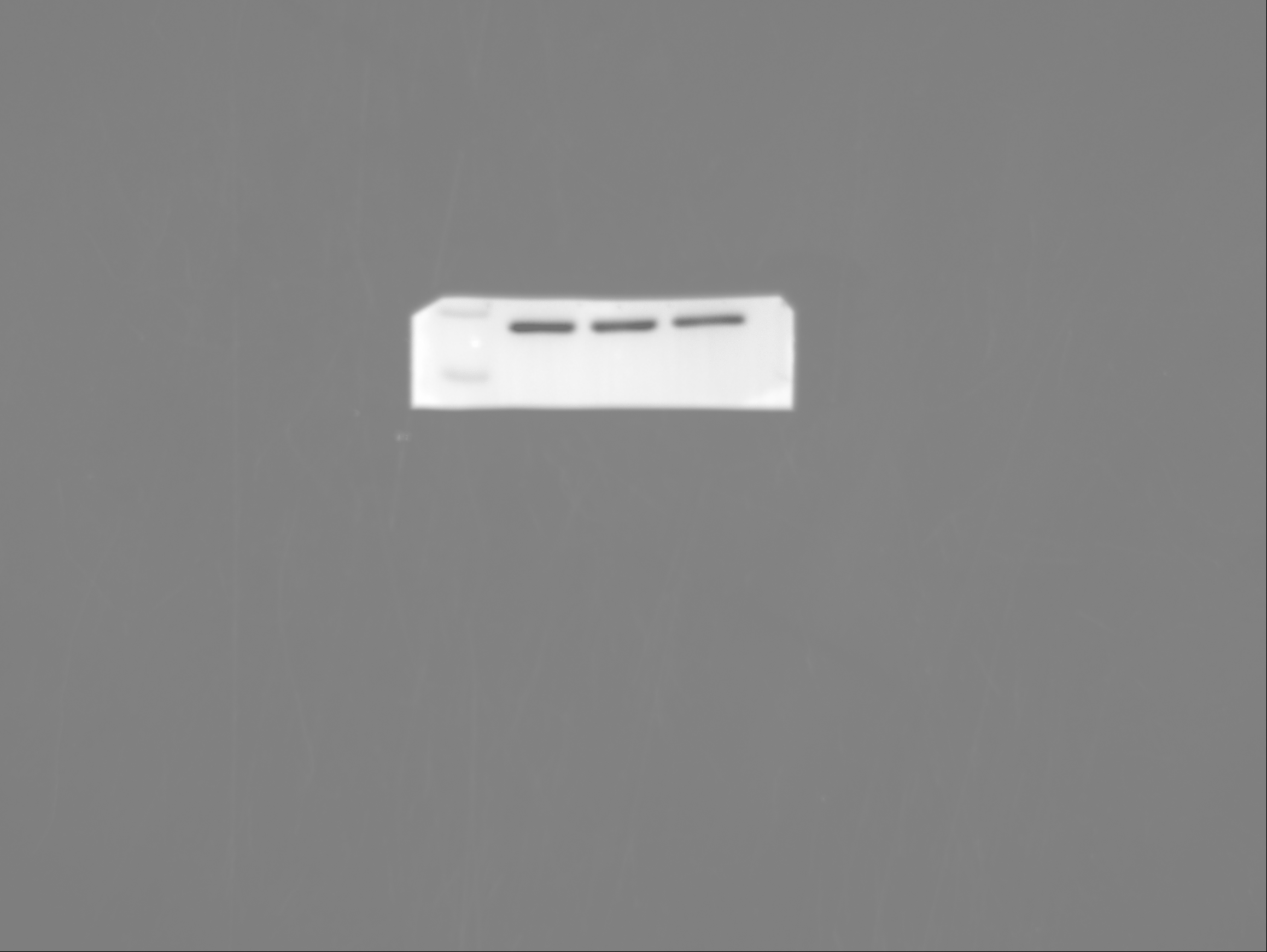

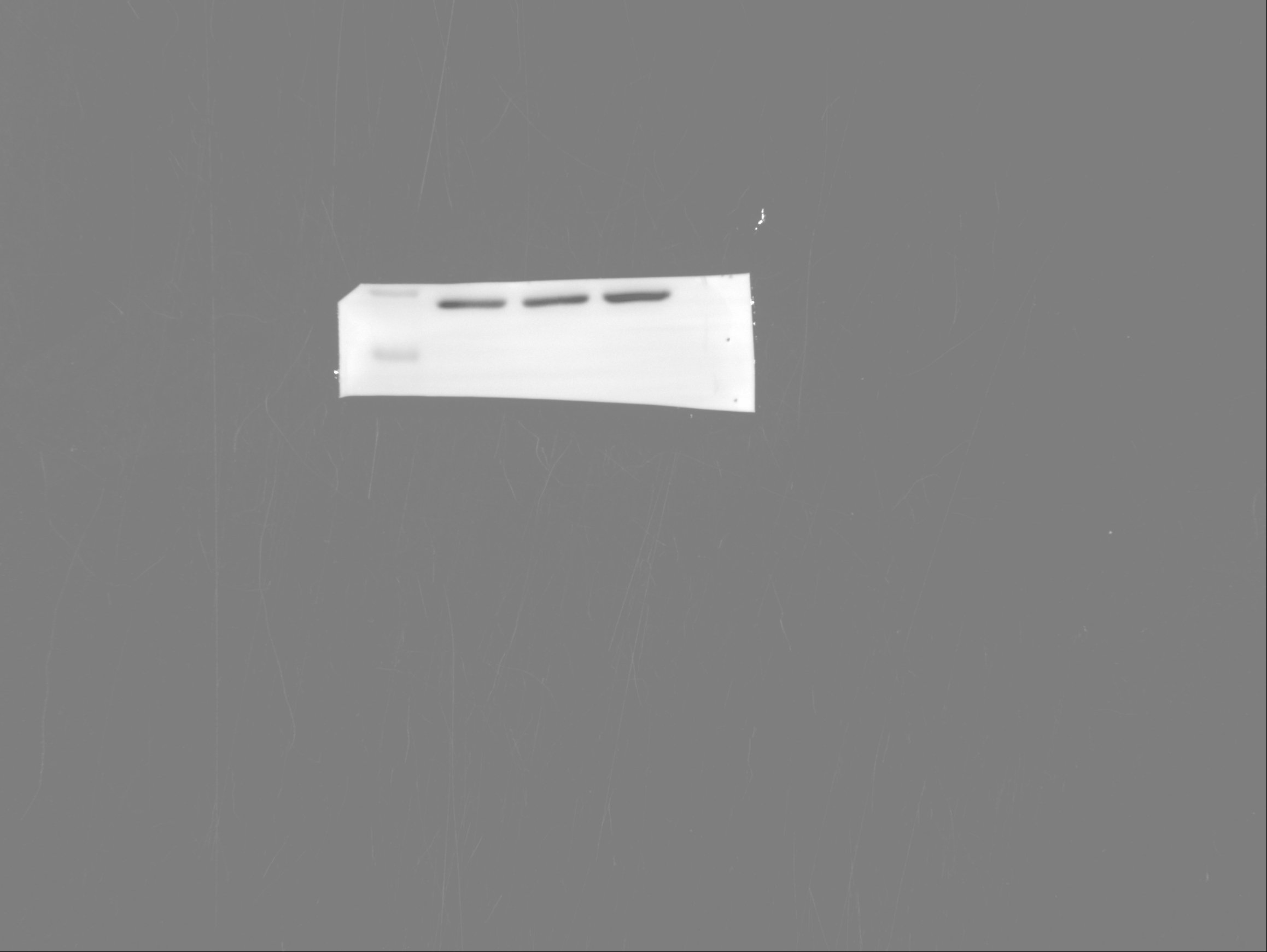


P21

GAPDH

GAPDH


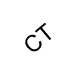

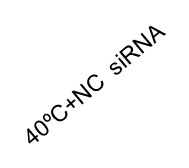

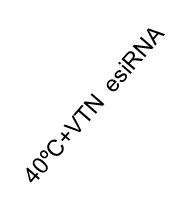

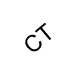

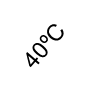

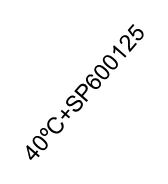


c

d

**Supplementary Figure S3:**


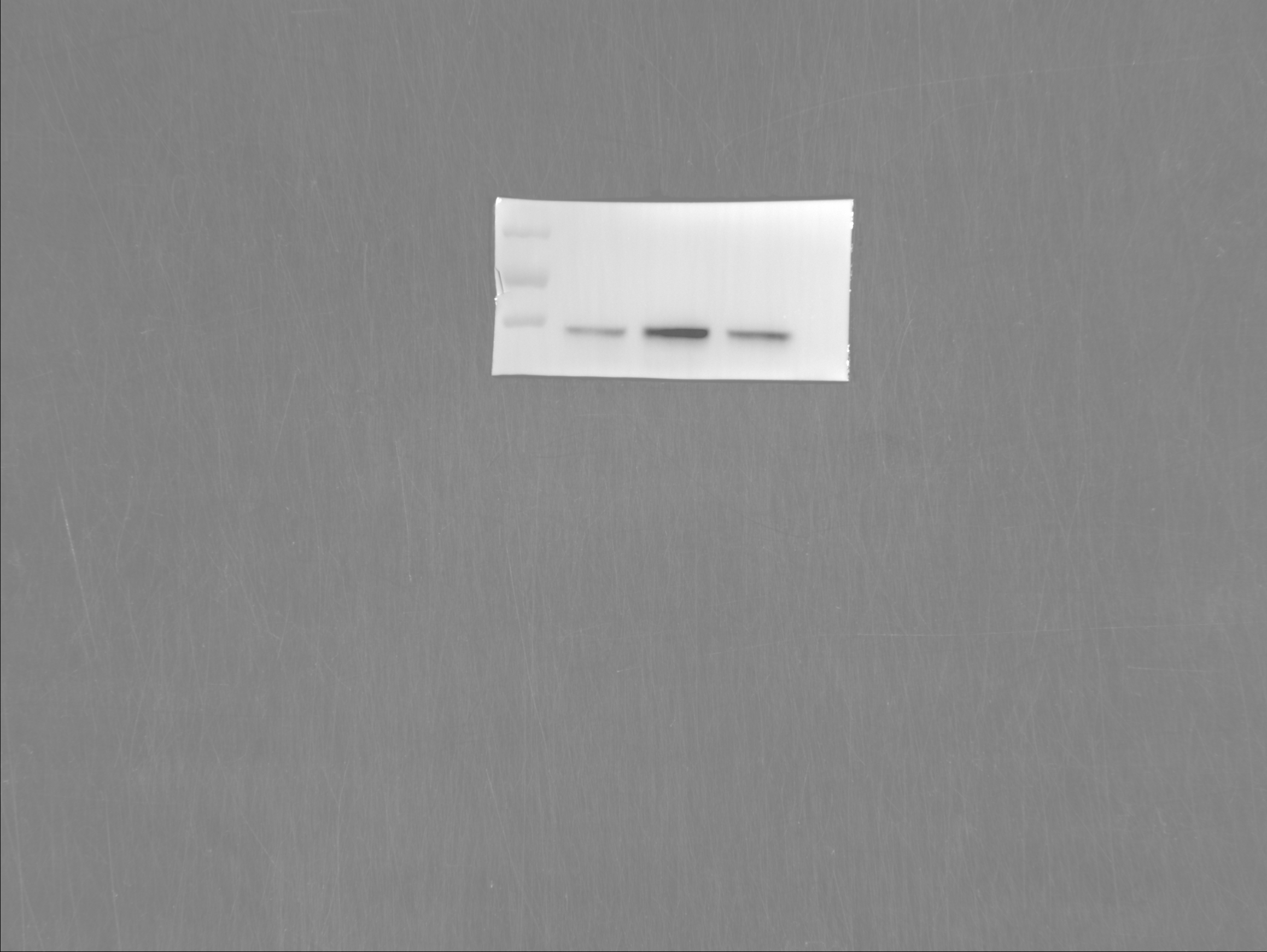

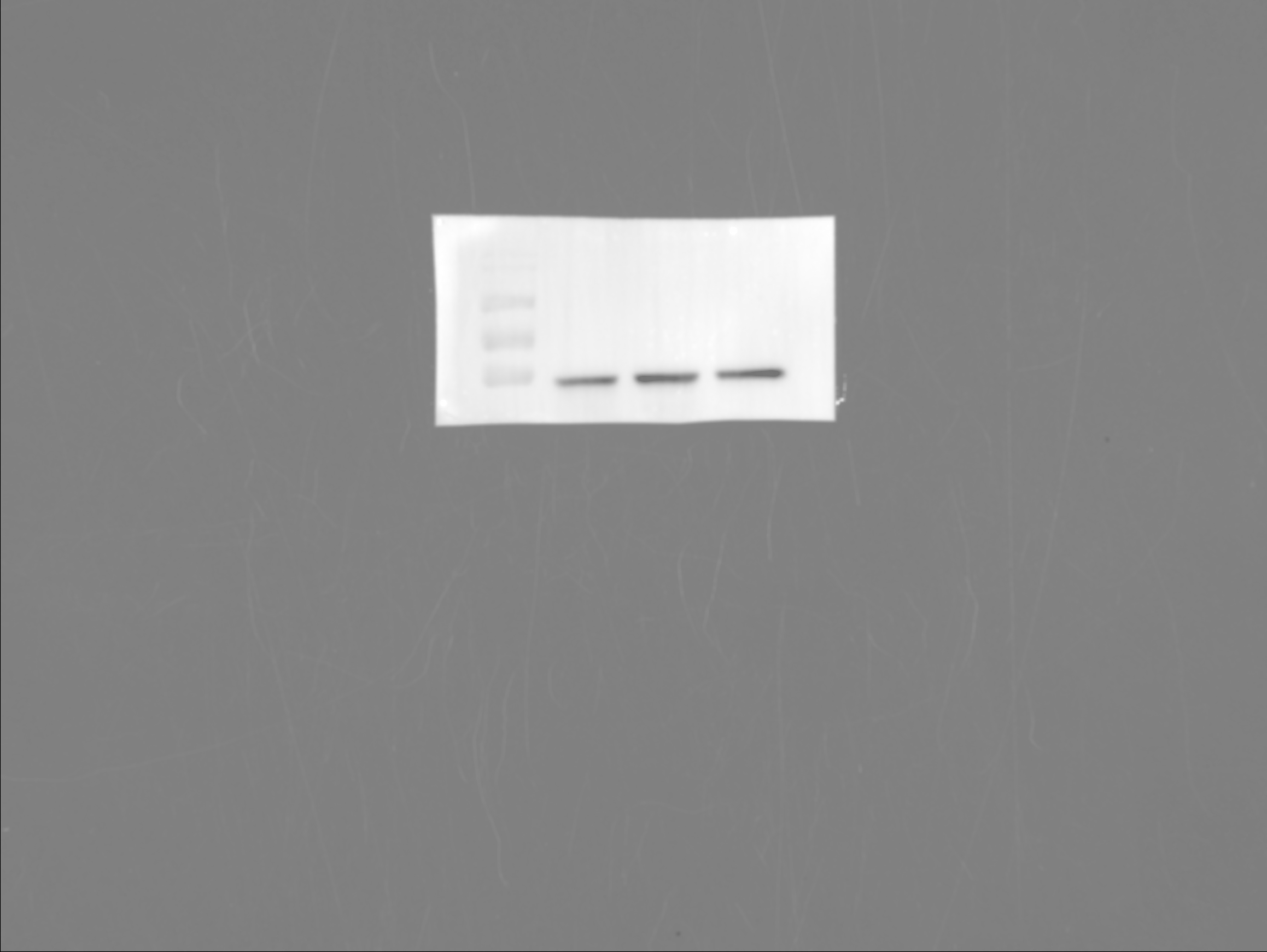


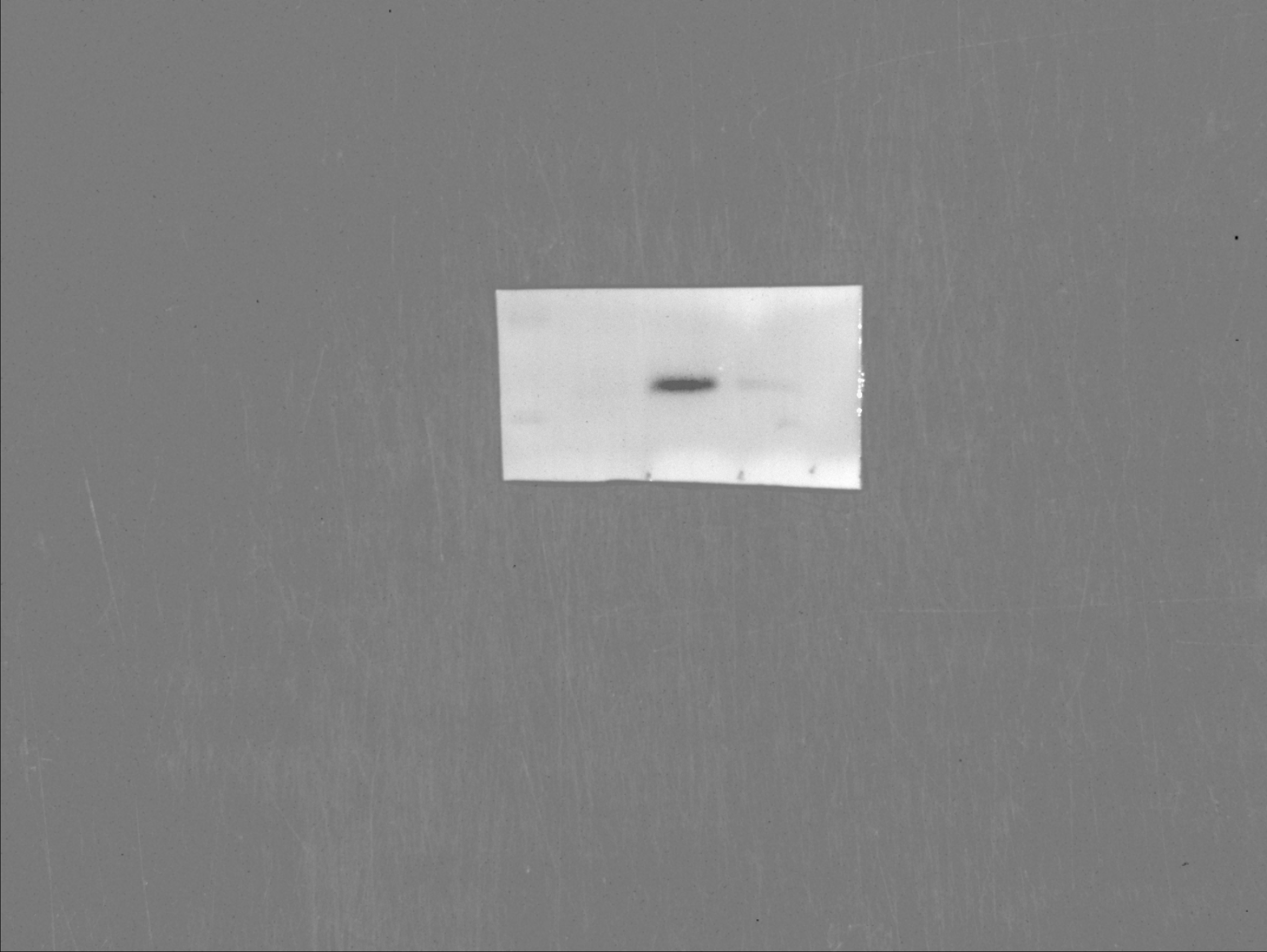

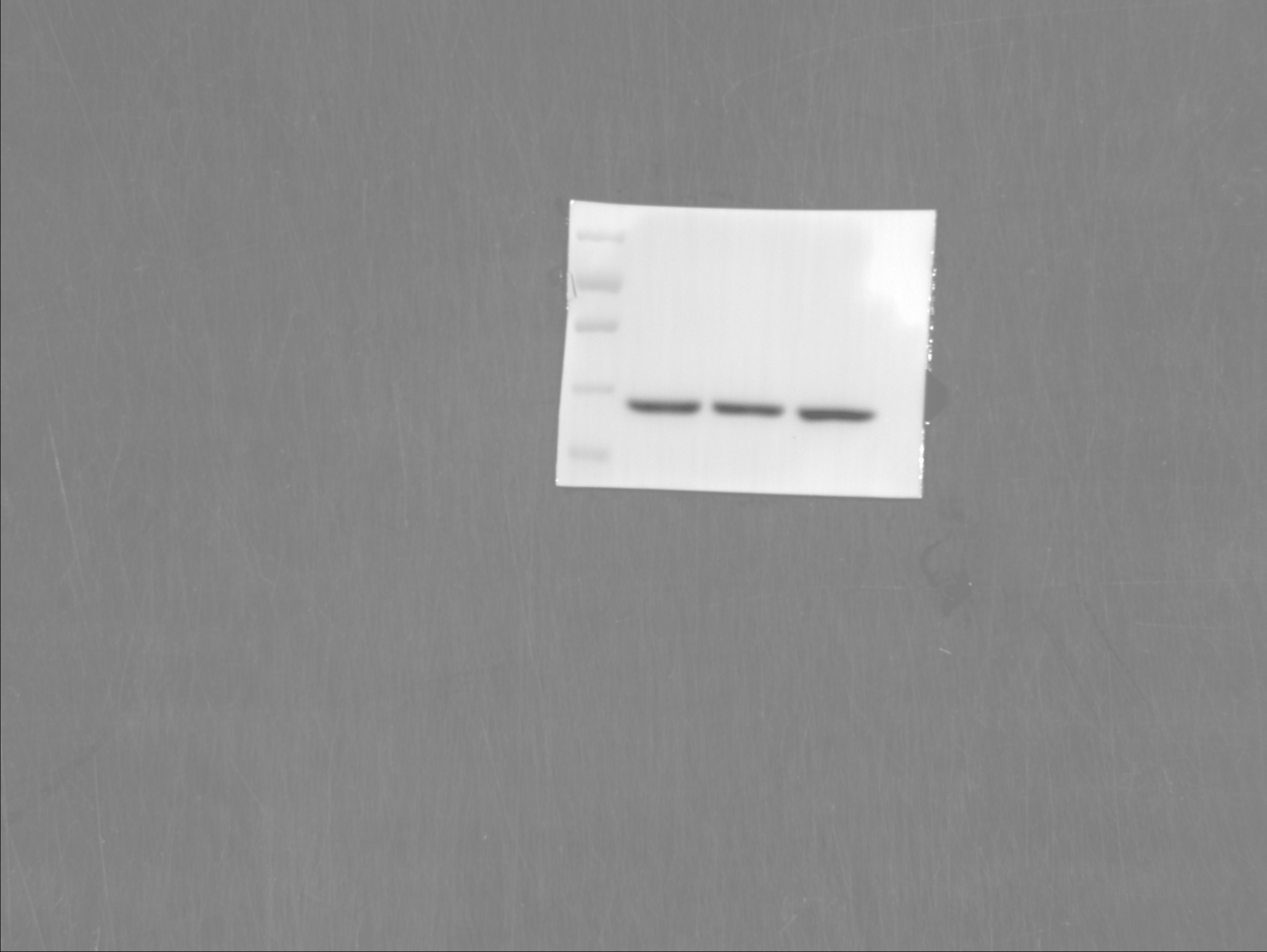

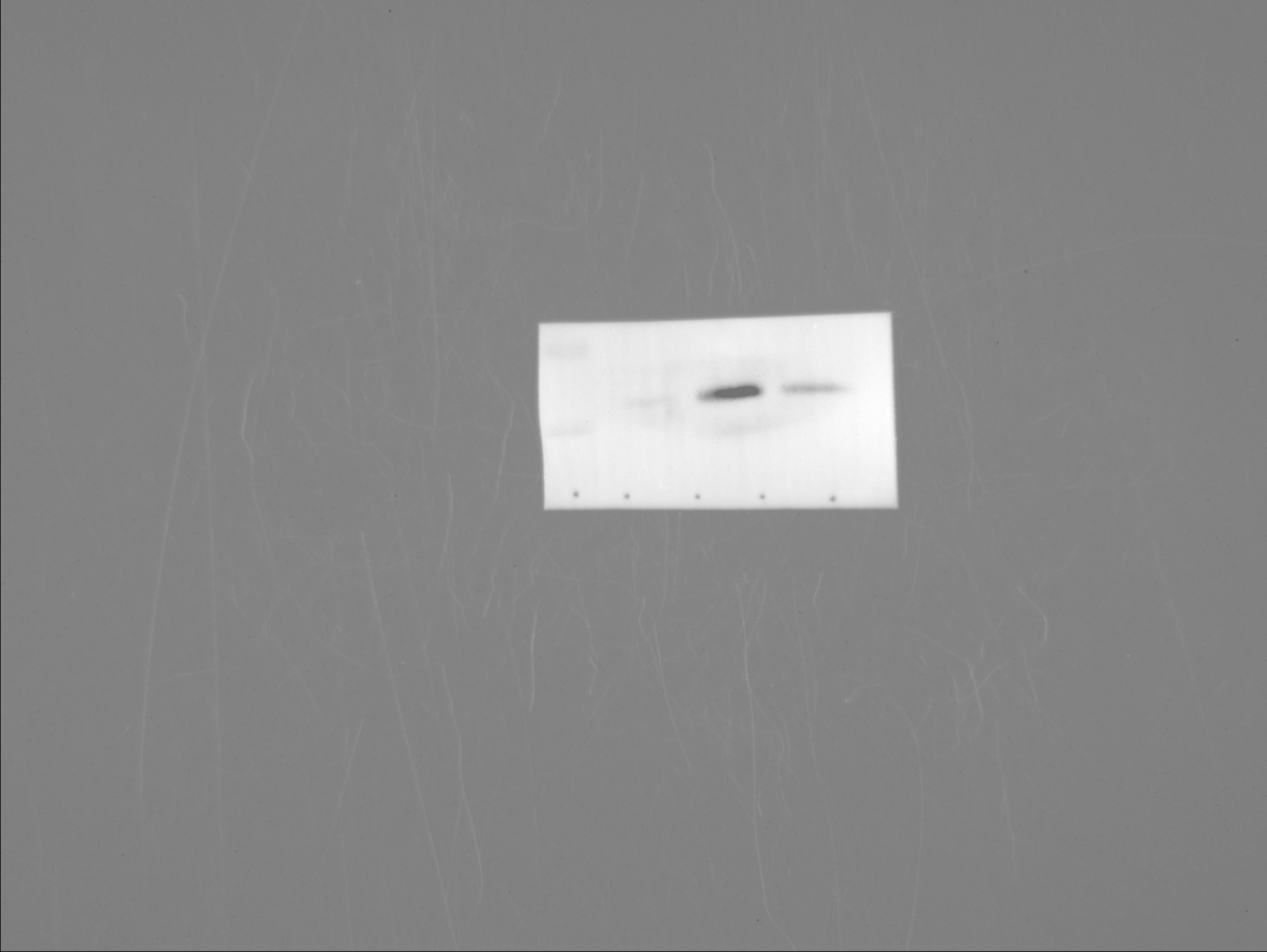

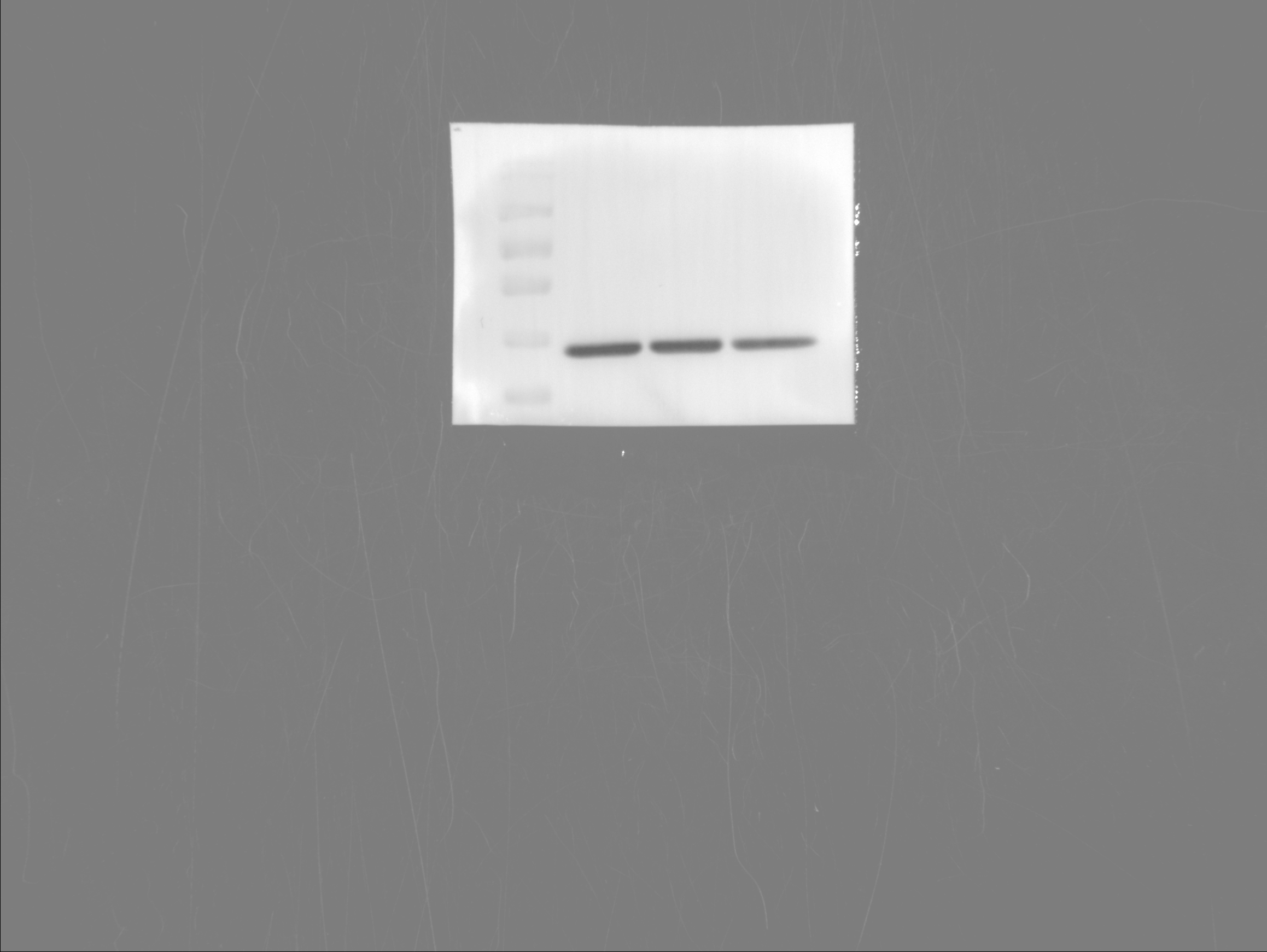


P21

GAPDH

P53

P53

P21

GAPDH

Supplementary Figure S3:

Original Western blot images from Fig 4f (a), Fig 5a (b), Fig 6f (c) and Fig 6l (d).
